# A Novel Primer Mixture for GH48 Genes: Quantification and Identification of Truly Cellulolytic Bacteria in Biogas Fermenters

**DOI:** 10.3390/microorganisms8091297

**Published:** 2020-08-25

**Authors:** Regina Rettenmaier, Yat Kei Lo, Larissa Schmidt, Bernhard Munk, Ilias Lagkouvardos, Klaus Neuhaus, Wolfgang Schwarz, Wolfgang Liebl, Vladimir Zverlov

**Affiliations:** 1 Chair of Microbiology, Technical University of Munich, Emil-Ramann-Str. 4, 85354 Freising, Germany; regina.rettenmaier@tum.de (R.R.); kei.lo@campus.lmu.de (Y.K.L.); schmidt_larissa@mail.ru (L.S.); wliebl@wzw.tum.de (W.L.); 2 Bavarian State Research Center for Agriculture, Central Department for Quality Assurance and Analytics, Lange Point 6, 85354 Freising, Germany; bernhard.munk@lfl.bayern.de; 3 ZIEL—Core Facility Microbiome, Technical University of Munich, Weihenstephaner Berg 3, 85354 Freising, Germany; ilias.lagkouvardos@tum.de (I.L.); neuhaus@tum.de (K.N.); 4 Aspratis GmbH. Munich, Germany, Hübnerstr. 11, 80637 Munich, Germany; schwarz@outlook.de; 5 Institute of Molecular Genetics of National Research Centre (Kurchatov Institute), Kurchatov Sq. 2, 123182 Moscow, Russia

**Keywords:** amplicon sequencing, qPCR, biomass hydrolysis, taxonomy, DNA, RNA

## Abstract

Genomic studies revealed the glycoside hydrolases of family 48 (GH48) as a powerful marker for the identification of truly cellulolytic bacteria. Here we report an improved method for detecting cellulolytic bacteria in lab-scale biogas fermenters by using GH48 genes as a molecular marker in DNA and RNA samples. We developed a mixture of primers for the specific amplification of a GH48 gene region in a broad range of bacteria. Additionally, we built a manually curated reference database containing GH48 gene sequences directly linked to the corresponding taxonomic information. Phylogenetic correlation analysis of GH48 to 16S rRNA gene sequences revealed that GH48 gene sequences with 94% identity belong with high confidence to the same genus. Applying this analysis, GH48 amplicon reads revealed that at mesophilic fermenter conditions, 50–99% of the OTUs appear to belong to novel taxa. In contrast, at thermophilic conditions, GH48 gene sequences from the genus *Hungateiclostridium* dominated with 60–91% relative abundance. The novel primer combinations enabled detection and relative quantification of a wide spectrum of GH48 genes in cellulolytic microbial communities. Deep phylogenetic correlation analysis and a simplified taxonomic identification with the novel database facilitate identification of cellulolytic organisms, including the detection of novel taxa in biogas fermenters.

## 1. Introduction

One of the primary targets in bioeconomy is the production of biofuels such as biodiesel, bioethanol and biogas [1]. Crops like maize (whole plant) have been widely utilized for the production of biogas via anaerobic digestion. Lignocellulose—the fibrous material in the plant cell wall—accounts for 60% of total biomass on earth. Depending on its source, plant leaves and stems consist of lignocellulose: 35–50% cellulose, 20–35% hemicellulose, 10–25% lignin and small amounts of other components [2]. In biogas fermenters, the lignocellulosic substrate passes through several steps: (i) saccharolytic bacteria hydrolyze the polysaccharide components of biomass to soluble oligo- and monosaccharides; (ii) acidogenic and acetogenic bacteria use the sugars and products from primary fermentation to produce mainly acetate, hydrogen and carbon dioxide; (iii) methanogenic archaea produce methane and carbon dioxide [3]. The first step in biomass fermentation, which is mainly driven by carbohydrate active enzymes (CAZymes), can be a bottleneck due to the recalcitrant parts of biomass such as highly lignified material or cellulose fibers with a high degree of crystallinity [4,5,6].

The application of high-throughput sequencing technologies enabled a deeper insight into the biogas microbiota [7]. In general, 16S rRNA gene sequences are used for the characterization of microbial communities [8,9,10], but the resolution of this method is limited especially when only shortened sequences (i.e., amplicons) of the gene are targeted [11]. In addition, 16S rRNA genes are inferior when functional groups of organisms are to be analyzed. Due to considerable functional variability within the taxa, taxonomic identification per se does not provide reliable information about the metabolic properties of an organism [12,13,14,15,16]. For this purpose, characterization of functional genes is advantageous allowing inferences about the function of interest. To this end, genes or transcripts of key enzymes of metabolic pathways can serve as a representative marker for a pathway of interest and the linked physiological features.

For the solubilization of crystalline cellulose, in bacteria the glycoside hydrolases of the hydrolytic enzyme families 5, 6, 8, 9 and 48 are crucial [17]. Remarkably, as far as studied to date, all organisms possessing at least one glycoside hydrolase of family 48 (GH48) are truly cellulolytic, i.e., can substantially degrade crystalline cellulose [17,18]. In contrast, most bacteria producing GH5 and GH9 enzymes can hydrolyze soluble (artificial) cellulose substrates such as carboxymethyl cellulose. However, they are unable to degrade crystalline cellulose. This makes GH48 a suitable molecular marker for the characterization of truly cellulolytic bacteria especially in anaerobic environments [14,16,19].

However, previous studies on GH48 gene sequences are based on genes known at their time. The steadily growing availability of metagenomes and an increasing number of bacterial genome sequences has led to a dramatic increase of identified GH48 gene sequences in public databases [20,21]. Despite this, there are only a limited number of open-access databases focusing on carbohydrate-active enzymes (CAZymes) like GHs. The CAZy database (http://www.cazy.org/) [21] is currently the most complete database documenting over 100,000 non-redundant CAZymes. Nevertheless, this database only provides protein sequences of GH48, which hampers gene analysis. The GenBank database at the National Center for Biotechnology Information (NCBI) contains about 26 bacterial GH48 enzymes from 9 bacterial genera with links to the corresponding nucleotide sequences. Yet, GH48 is known to be expressed by at least 50 genera [21,22]. Thus, an analysis solely based on the latter genes does not reflect the phylogenetic diversity of GH48, not to mention the genes in hitherto unknown species.

To fill this gap, a dedicated GH48 database composed of GH48 coding nucleotide sequences, the corresponding protein sequences, as well as the affiliated 16S rRNA gene sequences and corresponding taxonomy was constructed. So far, it proved not possible to develop a nucleotide primer pair for detecting and amplifying a broad range of GH48 genes [14,16,23]. To monitor as many GH48 gene sequences in a given metagenomic or metatranscriptomic sample as possible, the primers used for amplification should cover a broad range of variants. To this end, we improved GH48 as a molecular marker system for the identification of cellulolytic key players via GH48-amplicon sequencing. The developed novel primers were used to amplify GH48 gene sequences from DNA and RNA isolated from lab-scale biogas fermenters at four different process conditions. The identification of cellulolytic organisms in such fermenters helps to shed light on the microbial diversity at the specific fermenter stage and performance and thereby to come to a better understanding of the ‘black box’—biogas microbiome [7,24,25]. In addition, these four process conditions were analyzed to study the relative abundance of truly cellulolytic bacteria compared to the total copy number of bacterial 16S rRNA genes by using qPCR for quantification of the GH48 and 16S rRNA genes. The quantification of the latter genes can help to assess the importance of the first step in the overall process of microbial conversion of biomass to biogas.

## 2. Materials and Methods

### 2.1. Primer Development

Based on 87 bacterial GH48 gene sequences (Supplementary Material Table S1) and previous primers [14], a new mixture of 16 forward and nine reverse primers, designated cel48-Mix2, was developed. The GH48 gene sequences were aligned to the sequence of *Hungateiclostridium thermocellum* (ABN53296.1), formerly known as *Clostridium thermocellum,* to identify possible primer positions in each sequence. Chosen primer binding sites were aligned again individually and resulting groups were manually combined by introducing degenerate bases or deoxy-inosine [I]. Degenerate positions were used only if this increased number of combined species. The primer mix cel48-Mix2 and cel48_490F_I & cel48_920R_I (modified after Pereyra et al., 2010 [14]; see Table 1 and Table 2) were tested for specificity and phylogenetic diversity. Amplicons were cloned using TOPO TA Cloning^®^ pCR4 in *Escherichia coli* TOP10 (both Invitrogen, Carlsbad, CA, USA) and sequenced (Eurofins MWG). Resulting sequences, without primer, were aligned (ClustalW in MEGA7 [26]). To assess the phylogenetic diversity, Maximum-likelihood trees based on the Tamura–Nei model [27] with 1000 bootstraps were calculated for diversity assessment and sequences were compared to the NCBI database using BLASTx.

### 2.2. Nucleic Acid Extraction from Biogas Fermenter Samples

Total DNA and RNA was extracted from digestate (GR from German: *Gärrest*) of lab-scale biogas fermenters fed with maize silage and operated at 38 °C (mesophilic, M) or 52–55 °C (thermophilic, T) at the Bavarian State Research Center for Agriculture (Freising, Germany). At either mesophilic or thermophilic reactor operation, different fermentation stabilities and performances were tested: ‘stable’ with no accumulation of volatile fatty acids and methane yield of 350–400 L_STP_ CH_4_ × kg_VS_^−1^ or ‘instable’ with acidification due to the accumulation of volatile fatty acids and reduced methane yield). For the fermenter stages analyzed (mesophilic-stable, MS; mesophilic-instable, MI; thermophilic-stable, TS; thermophilic-instable, TI.), see Supplementary Material Table S2. Next, *in sacco* (IS) enrichment, originally developed by Mohamed et al. [30], was performed to enrich cellulolytic bacteria that adhere to the cellulose fibers directly in their natural habitat. *In sacco* was conducted at each fermenter stage as described previously [31] with slight modifications: Briefly, nylon bags (ANKOM Technology, Macedon, NY, USA) with a size of 5 × 10 cm and a pore width of 50 μm were filled with 2 g shredded filter paper (Whatman No. 1), fixed on a custom-made sample support device and directly submersed into the lab-scale biogas fermenter. Two biological replicates were incubated for 5 days. Each *in sacco* sample was carefully washed with isotonic NaCl solution (0.9% (*w*/*v*)) to remove remaining digestate. The washed remains were used for DNA and RNA extraction with the EURx GeneMATRIX DNA + RNA + protein purification kit (Roboklon, Berlin, Germany) with cell lysis modifications as described in Rettenmaier et al., 2020 [32]. In brief, cell lysis was achieved by adding 300 µL ROTI-phenol (Carl-Roth, Karlsruhe, Germany) to 300 mg sample in a lysing tube with lysing matrix E (MP Biomedicals, Eschewege Germany) and bead-beating for 2 × 20 s at 5 m/s followed by centrifugation for 10 min at 10,000× *g*. Hundred microliter of clear supernatant were mixed with 200 µL Lyse ALL buffer and 300 µL DRP buffer and applied to the DNA-binding column (Roboklon). Subsequent purification of nucleic acids was performed following the manufacturer’s instructions. The DNA was eluted in 40 µL elution buffer, RNA was eluted in 40 µL RNase-free water.

### 2.3. (Quantitative) Polymerase Chain Reaction (qPCR)

Primers used are summarized in Table 1. Performing PCR with degenerate primers poses certain challenges for DNA polymerases. For successful amplification of GH48 gene sequences with cel48-Mix2, it was essential to use platinum *Taq* DNA polymerase (Thermo Fisher, Waltham, MA, USA). (q)PCRs were found specific and efficient when using 6-mM MgCl_2_ and 2-µM primer mix each. Each 25-µL reaction (adjusted with water) contained 2.5 µL 10xTaq Buffer; 3 µL MgCl_2_ (50 mM); 0.5 µL dNTPs (10 mM each); 0.5 µL cel48-Mix2F (100 pmol/µL); 0.5 µL cel48-Mix2R (100 pmol/µL); 0.2 µL Platinum *Taq* DNA polymerase. The amount of template varied from 5–50 ng. For qPCR, 1 µL of EvaGreen^®^ (Biotium, Hayward, CA, USA) was added. The PCR was conducted as follows: initial denaturation for 10 min at 95 °C, followed by 26 or 40 cycles (for NGS or qPCR, respectively) of 95 °C for 20 s; 60 °C for 20 s and 72 °C for 20 s followed by a final elongation step for 2 min at 72 °C. For qPCR of 16S rRNA genes, a two-step PCR was applied (40 cycles 15 s at 95 °C and 1 min at 70 °C). To avoid quantification of primer-dimers in (RT)-qPCR of both 16S rRNA and GH48 genes, an additional step for 10 s at 83 °C after elongation was applied. Quantification by fluorescence was measured immediately after this step (Supplementary Material Figure S1). Dissociation curves of amplicons were measured using EvaGreen as follows: 1 min at 95 °C, 30 s at 55 °C and 30 s at 95 °C using increments of 1 °C up to 95 °C.

### 2.4. Reverse Transcription (RT)

Total RNA was cleaned from residual DNA with DNase (Turbo DNA-*free*^TM^ kit, Ambion, Austin, TX, USA) following manufacturer’s instructions. Gene-specific RT was performed using 5 µL cleaned RNA, 0.5 µL reverse primer (10 pmol/µL) and adjusted to 8 µL with DEPC-water (Invitrogen). The mixture was incubated 5 min at 65 °C and 10 min on ice. For GH48 genes, primer cel48_920R_I [14] or cel48-Mix2R and for 16S rRNA genes, primer R1378_mod [33] were used. RT was performed by adding 2 µL dNTP-mix (10 mM of each nucleotide), 2 µL DTT, 2 µL buffer and 1 µL reverse transcriptase (Affinity Script Multi Temp, Agilent, Santa Clara, CA, USA) and incubation for 1 h at 45 °C, followed by 15 min at 70 °C. Negative RT-qPCR controls without reverse transcriptase were performed for each RNA extraction.

### 2.5. DNA Standard for Absolute Quantification

For the quantification of GH48 gene sequences, a plasmid DNA standard with *cel48S* of *H. thermocellum* cloned into pET24c was chosen (TU Munich, München, Germany). Plasmids were extracted from *E. coli* DH10β cells via the QIAGEN Plasmid Midi Kit, following the manufacturer’s instructions and linearized with *Nde*I (Thermo Fisher). Genomic DNA of *E. coli* DH10β, extracted from a cell pellet of a 50 mL overnight culture, was used as background DNA in the qPCR standard. For extraction of genomic DNA, the cell pellet was resuspended in 5 mL lysing buffer (200-mM Tris/HCl; pH 7.5, 35 mM EDTA, 75 mM NaCl, 1 mg/mL lysozyme) and incubated 1 h at 37 °C followed by 2 h at room temperature after addition of 500 µL SDS (10% *w/v*) and 500 µL proteinase K (1 mg/mL; AppliChem, Darmstadt, Germany). For removal of proteins, 2 mL of 5 M NaCl and 1/3 of the total volume chloroform/isoamyl alcohol (24:1, Carl-Roth) were added. The mixture was shaken (20 min, 180 rpm, room temperature) and centrifuged (10 min, 5000× *g*, room temperature). The purified DNA was precipitated from the supernatant with one volume isopropanol, centrifuged, washed with ethanol (70% *v/v*) and finally dissolved in 200 µL ddH_2_O. The final qPCR standard consisted of 50 ng/µL gDNA of *E. coli* with a serial dilution of 10^3^–10^9^ GH48 gene copies/µL. The number of GH48 gene copies was calculated from the concentration of the linearized plasmid DNA as well as the molecular weight based on the nucleotide sequence (Available online: http://www.bioinformatics.org/sms2/dna_mw.html). For the quantification of 16SrRNA gene sequences, genomic DNA of *E. coli* was used in serial dilutions as standard. The number of 16S rRNA gene sequences in the standard reaction was assessed via MPN-qPCR.

### 2.6. GH48 Reference Database Construction

To link the GH48 sequences to taxonomic units, nucleotide sequences of GH48 and 16S rRNA gene sequences from the same species were required. Thus, to generate the database, ‘glycosyl hydrolase family 48′ was searched in NCBI RefSeq. All entries found, together with a GenPept file (i.e., full protein description), were downloaded. The taxonomy identification (txid) was retrieved from the GenPept files using a custom script (GenPept2txid.py, Supplementary Material Script S1). Genome assemblies (complete genome, chromosome, scaffold and contig) with RefSeq annotations were downloaded from the NCBI Assembly database using the txid with Batch Entrez [34]. From the GenBank files, features (species name, nucleotide sequence, protein sequences, accession number and gene product name) were extracted using the custom script Genbank2Features.py (Supplementary Material Script S2), which searches for genome assemblies with one of the protein accession numbers of interest. 16S rRNA gene sequences were extracted from the same GenBank using the custom script Genbank216S.py (Supplementary Material Script S3). Nucleotide sequences of the 16S rRNA genes are extracted from the GenBank files annotated as ‘16S ribosomal RNA’. For species with more than one 16S rRNA gene, the first found was chosen. Taxonomy was retrieved using the txid with taxonomizr (Available online: https://github.com/sherrillmix/taxonomizr/). All information was combined using R (Available online: https://www.r-project.org/). For species and strains with more than one genome assemblies, only information extracted from one of the assemblies was retained. Identical sequences, GH48 modules or amplicons, in between different strains, species or genera were identified by clustering sequences at 100% identity using USEARCH [35]. The final database (‘combined database’) was exported as an Excel file (Supplementary Material File S1). All GH48-module sequences of this database are available as FASTA file (Supplementary Material File S2).

### 2.7. Phylogenetic Correlation of 16S rRNA Genes to GH48 Gene Sequences

GH48 genes are used as a molecular marker either full length (1800–2100 bp) or using a 344-bp amplicon [14]. For identity thresholds based on GH48, 16S rRNA gene identities were taken as base and correlated to GH48. For GH48-amplicon thresholds, either the genus identification or operational taxonomic unit (OTU) clustering at genus level was used. Sequences from the ‘Combined database’ were extracted into GH48-16S and GH48-16S-C datasets. For the former, GH48 amplicon and the corresponding full-length 16S rRNA sequences of any source were combined into the GH48-16S dataset; for the latter, GH48 amplicon and full-length 16S rRNA sequences extracted only from complete genomes were combined into the GH48-16S-C dataset. The amplicon sequences were extracted from full-length sequences in the ‘Combined database’ by aligning reference amplicon sequences using MUSCLE [36] and extracting the aligned region using MEGA7 [26]. Since the ‘Combined database’ was dominated by *Streptomyces*, such without valid species name were excluded from GH48-16S, but not from GH48-16S-C, since for the latter *Streptomyces* accounted for a smaller proportion. From both datasets, hypothetical proteins were also excluded.

To model the sequence identity correlation, GH48 amplicon sequences extracted and full-length 16S rRNA gene sequences were aligned separately with MUSCLE [36] and distance matrices of the alignment were calculated using MOTHUR [37]. For each dataset, distance matrixes were combined using tidyverse (Available online: https://tidyverse.tidyverse.org/) and reshape2 (Available online: https://github.com/hadley/reshape) and plotted in R with ggplot2 (Available online: https://github.com/tidyverse/ggplot2). The corresponding GH48 sequence similarities to 16S rRNA similarities were determined by fitting a polynomial model and a linear model. To evaluate the modelling, adjusted R^2^ (R_adj^2), root-mean-square error (RMSE), mean absolute error (MAE) and predicted residual error sum of squares (PRESS) was computed in R using caret (Available online: https://github.com/topepo/caret) and DAAG (Available online: https://CRAN.R-project.org/package=DAAG).

Given that there is no universal agreement on 16S rRNA thresholds for species or genus classification, GH48 thresholds corresponding to 99%, 97% or 95% 16S rRNA gene sequence similarity, respectively, as proposed from several authors in the past years [11,38,39,40], may not accurately cluster reads from the same species or genus into the same OTU. To improve the reliability of the GH48 threshold, another threshold was computed based on taxonomic annotations in NCBI. To this end, GH48 amplicons and full-length module sequences were clustered at a range of sequence identity at 1% increment using USEARCH [35]. A clustering agreement was computed for each percentage of identity. The clustering agreement was defined as percentage of sequences belonging to the same genus or species (according to the taxonomic annotation in NCBI) that were being clustered into the same OTU. A 100% agreement indicated that all sequences were clustered into the correct genus or species.

### 2.8. Next-Generation-Sequencing (NGS) and Data Analysis

For sequencing with Illumina-MiSeq, adaptors were added to each primer within cel48-Mix2. Adaptor sequences are TCGTCGGCAGCGTCAGATGTGTATAAGAGACAG (forward) and GTCTCGTGGGCTCGGAGATGTGTATAAGAGACAG (reverse). PCR reactions were cleaned using Ampure beads in accordance with the manufacturer’s instructions and amplified in a second PCR reaction to attach sequencing adapters following Berry et al. [41]. Final libraries were pooled equimolarly and sequenced on a MiSeq system as recommended by the manufacturer (e.g., Reitmeier et al. [42]).

Data analysis of raw sequence reads was performed with an UPARSE-based analysis pipeline [43]. Demultiplexing was performed by demultiplexor_v3.pl (Perl script, available on request). Pairing, quality filtering and OTU clustering (94% identity) was done by USEARCH 8.0 [35]. Taxonomic classification was performed manually by pairwise sequence comparison via BLASTn with the database constructed within this work (see ‘2.5 GH48 reference database construction’). Sequence alignment was done by MUSCLE [36] and treeing by Fasttree [44]. The number of allowed mismatches in the barcode was 1. Minimal fastq quality score for trimming of unpaired reads was 20. Length for paired reads was set between 320–500 bp with a maximal rate of expected errors of 0.02 in paired sequences. Trimming at the forward and reverse side of the sequence was 10 bp. Statistical analysis was performed via Rhea [45] in R.

## 3. Results

### 3.1. Primer Development

In silico GH48 primer development resulted in 16 different, partly degenerated forward primers in combination with 9 different, partly degenerative reverse primers. The best composition of primers was experimentally verified (see ‘2.1 Primer development’ in Materials and Methods section) and resulted in the use of certain primer mixtures (Table 1 and Table 2). The calculation of the mixture composition was based on the amount of degenerative positions within a single primer. A degenerative position was defined as a position with two possible bases. The introduction of the non-natural base deoxy-inosine [I], which was preferred rather than introducing a degenerative position with three or four bases, was not regarded as a degenerative position in this context. The fraction of a single primer was calculated with the following formula: V = 2^N^, with N being the number of degenerations, see Table 2 for the detailed fractions of cel48-Mix2.

Comparison of the phylogenetic diversity of amplified GH48 gene sequences obtained with cel48-Mix2 or cel48_490F_I + cel48_920R_I [14] revealed strong overlaps. With both primer sets, nine different clusters of GH48 gene sequences were detected (Supplementary Material Figure S2 and Table S3). Although the primer design was only based on GH48 gene sequences of organisms with validly published species names, GH48 gene sequences with high homology to hitherto unknown bacteria were also amplified. Amplification via cel48-Mix2 led to the detection of new GH48 gene sequences with homology to *Paenibacillus* sp., which were not detected using cel48_490F_I and cel48_920R_I. Besides, for qPCR the cel48-Mix2 had also advantages compared to the formerly developed primers and a higher PCR efficiency was achieved (see ‘3.3 Quantification of GH48 and 16S rRNA gene sequences’ below). Subsequently, amplification of GH48 gene sequences via cel48-Mix2 was adjusted for the Illumina MiSeq sequencer to increase the sequencing depth of the molecular marker (see ‘3.6 GH48 amplicon sequencing’ below).

### 3.2. Isolation of Metagenomic DNA and Metatranscriptomic RNA

Metagenomic DNA and metatranscriptomic RNA was isolated from two different fermenters, operated at either 38 or 52–55 °C, at four or two different stages (i.e., volumetric loads), respectively (Supplementary Material Table S2). Extraction of nucleic acids was performed either from raw digestate (GR) or from *in sacco* (IS) enrichment samples (see ‘2.2 Nucleic acid extraction from biogas fermenter samples’ in Materials and Methods section), each in technical triplicates. Nucleic acids of the technical triplicates were pooled for further analysis (Supplementary Material Tables S4 and S5).

### 3.3. Quantification of GH48 and 16S rRNA Gene Sequences

Criteria for trustworthy absolute quantification are a sufficient PCR efficiency (85–125%) as well as a sufficient y-intercept (C_t_ values ranging from 35–40) [12]. For the quantification of GH48 gene sequences amplified with cel48-Mix2, a PCR efficiency of 93% was achieved (Supplementary Material Figure S3). Furthermore, the number of GH48 gene copies should not be over- or underestimated when using a plasmid-based GH48-gene standard as determined via the y-intercept (y-intercept: 37.7; Supplementary Material Figure S3). The PCR efficiency was lower (<90%) when the formerly published primers cel48_490F_I + cel48_920R_I [14] were used (data not shown). GH48 genes in the metagenome of 12 different samples derived from either GR or IS samples of the mesophilic or thermophilic biogas process (Supplementary Material Table S2) ranged from 9 × 10^4^ to 2 × 10^7^ gene copies/µL. Compared to the total amount of 16S rRNA genes, the overall GH48 gene copy numbers were low, ranging from 3–8 and 12–37 GH48 genes per 1000 rRNA genes in the DNA of GR or IS samples, respectively (Table 3 and Table 4 for GR or IS samples, respectively).

Interestingly, compared to the GH48 gene copy numbers obtained from analysis of the DNA samples, the sequence copy numbers were even lower in the RNA of the same fermenters. In some cases, no valid quantification for RNA samples was possible, as the copy numbers were below the linear detection limit of 10^3^ GH48 gene copies/µL (data not shown). This was true for the mesophilic (M) samples derived from stable (S) or instable (I) fermenter stages MS1-GR, MS2-GR and MI-IS or the thermophilic (T) sample, TS-GR. The ratio of GH48 genes to 16S rRNA genes quantified in cDNA of MS1-IS, MS2-IS, MS3-IS, MS3-GR, MI-GR, TS-IS, TI-IS and TI-GR ranged from 6 GH48 genes per 1,000,000 16S rRNA genes to 3 GH48 genes per 10,000 16S rRNA genes and was highest in experiment MS3-GR and lowest in experiment MS3-IS, respectively (Supplementary Material Table S6). For a detailed description of all fermenter-stage analyses, see ‘2.2 Nucleic acid extraction from biogas fermenter samples’ in Materials and Methods section as well as Supplementary Material Table S2.

### 3.4. Reference Database Construction

The NCBI RefSeq protein database contains about 1200 bacterial GH48-module containing proteins, excluding proteins annotated as “multispecies”. All entries with a definite taxonomic identifier were downloaded from the NCBI Assembly database (146 complete genome assemblies, 22 chromosome assemblies, 402 scaffold assemblies and 484 contig assemblies, state 20-05-2019). The final ‘combined’ database for GH48 genes contains 944 entries. Each entry consists of the taxonomic identifier (txid), organism name, nucleotide accession number, nucleotide sequence of the GH48-module containing protein, protein accession number, protein sequence, product name of the protein, 16S rRNA gene sequence and the expanded taxonomy of the organism. The coding sequences of GH48 modules range from 1429–7032 bp in our database.

The 944 entries mentioned above originate from 813 different species and strains belonging to 104 different genera of 42 bacterial families, while most the entries belongs to the genera *Streptomyces* and *Paenibacillus*. For a complete list of genera, see Supplementary Material Table S7. Out of 944 entries, 580 entries (61%) contained full-length 16S rRNA gene sequences (about 1500 bp). Two full-length 16S rRNA gene sequences contained ambiguous bases. Forty-five or 109 pairs with identical GH48-module nucleotide sequences or identical theoretical GH48 amplicon sequences (average length: 344 bp) were observed, respectively. Almost all pairs of identical GH48-module sequences belonged to strains of the same species, i.e., *Hungateiclostridium thermocellum* (former *Clostridium thermocellum*) DSM 1313 (ADU75731.1) and *H. thermocellum* AD2 (ALX09761.1) with an exception for *Phytoactinopolyspora* sp. YIM 96934 (WP_112256909.1) and *Actinobacteria* bacterium YIM 96077 (AYY11565.1), which probably belong to the same species as an analysis of the total genome sequence suggests (data not shown). Only in rare cases, identical amplicon sequences classified as GH48 were observed between different species of one genus, i.e., *Streptomyces malaysiensis* (WP_099013847.1) and *S. autolyticus* (AQA12037.1). The parameters of the database are summarized in Table 5, detailed information about the amplicon length distribution is shown as Supplementary Material Figure S4.

### 3.5. Correlation of 16S rRNA to GH48 Gene Sequences

**Figure 1:**
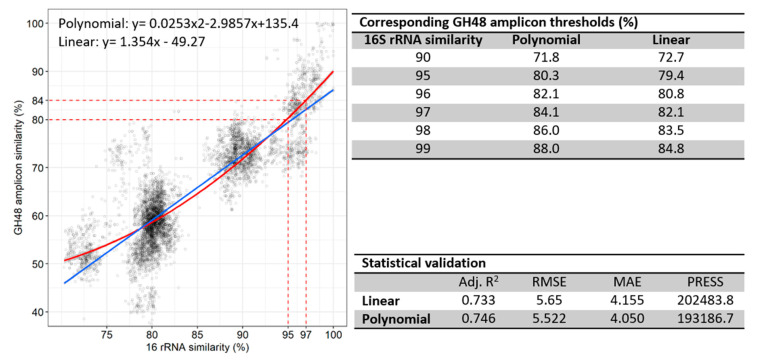
GH48 thresholds corresponding to 16S rRNA gene sequence similarity determined from 113 amplicons and full-length 16S rRNA gene sequences extracted from complete genome assemblies (GH48-16S-C subdataset). The corresponding GH48 sequence similarity to 16S rRNA similarities data were plotted by fitting a polynomial model (red) and a linear model (blue). To evaluate the modeling, adjusted R^2^, root-mean-square error (RMSE), mean absolute error (MAE) and predicted residual error sum of squares (PRESS) are shown.

16S rRNA sequences are widely utilized for OTU clustering and species identification based on specific sequence similarity [7,38]. To establish GH48 as a specific molecular marker for identification and taxonomic classification of truly cellulolytic bacteria, GH48 sequence similarity corresponding to certain 16S rRNA gene sequence similarity was computed using the subset GH48-16S-C (see: ‘2.5 GH48 database construction’ Materials and Methods section). This dataset contains GH48 amplicons and full-length 16S rRNA gene sequences from complete genome assemblies only, excluding hypothetical genes. The GH48 amplicon thresholds obtained from the GH48-16S-C dataset corresponding to 95%, 97% and 99% rRNA sequence identity were estimated to be 79%, 82% and 85% sequence identity, respectively, according to the linear model with adjusted R^2^ = 0.733, RMSE = 5.654, MAE = 4.155 and PRESS = 2,024,834 or 80%, 84% and 88% sequence identity, respectively, according to polynomial model with adjusted R^2^ = 0.746, RMSE = 5.522, MAE = 4.050 and PRESS = 193,187 (Figure 1). For simplification, data for the subset GH48-16S (see Materials and Methods: ‘2.5 GH48 database construction’), which resulted in similar values, are not shown.

**Figure 2:**
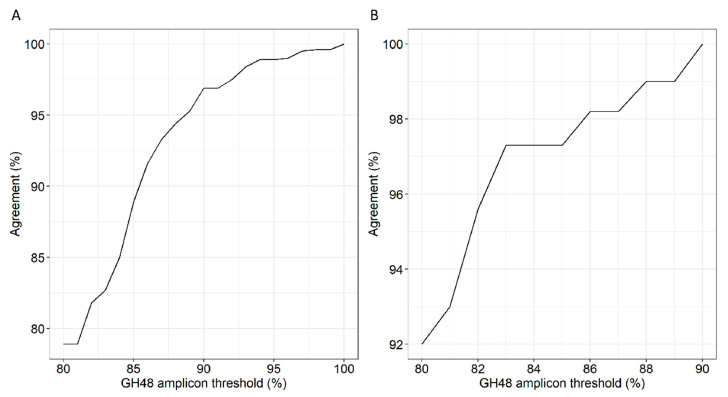
GH48 sequences clustering agreement with National Center for Biotechnology Information (NCBI) taxonomy. GH48 nucleotide sequences were clustered at a range of sequence identity at 1% increment using USEARCH [35]. (**A**) Clustering agreement of amplicon sequences based on GH48-16S dataset. All sequences were correctly clustered at genus level at 100% sequence identity; (**B**) Clustering agreement of amplicon sequences based on GH48-16S-C dataset. All sequences were correctly clustered at genus level at 90% sequence identity.

To ensure that the GH48 thresholds defined from 16S rRNA similarity approximated genus boundaries, GH48 amplicons from the subsets GH48-16S and GH48-16S-C were clustered using a range of sequence identity values. Thresholds, which enabled clustering the GH48 sequences at genus and species level according to their taxonomic annotation, were identified. Based on the GH48-16S dataset, 100% amplicon sequence identity was required to cluster all sequences correctly into different genera (Figure 2A). However, only 85% of the amplicons were correctly clustered at a threshold of 84% for the amplicon sequence identity, which should correspond to the genus boundary at 97% 16S rRNA sequence identity (determined by means of pairwise alignments). In contrast, for GH48 amplicons extracted from complete genomes only (subset GH48-16S-C), 97.3% of the sequences were correctly clustered at the proposed genus boundary of 84%. Further, all sequences were correctly clustered into their correct genera at 90% for the GH48 amplicon sequence identity (Figure 2B). Sequence pairs misplaced while clustering shared 94.2% to 98.2% 16S rRNA gene sequence identity. Assuming that these species may be misclassified in the database, these outliers were excluded. Thus, 94% amplicon sequence identity was required to classify all amplicon sequences correctly and this was chosen as threshold for any subsequent analysis.

### 3.6. GH48 Gene Amplicon Sequencing

To demonstrate the applicability of GH48 sequencing for species identification, the composition of true cellulolytic bacteria in the metagenome of lab-scale biogas fermenters was investigated. Sequencing reads of GH48 gene amplicons, generated with the Illumina MiSeq sequencer, were clustered into different OTUs with 94% amplicon sequence identity as explained above.

**Figure 3:**
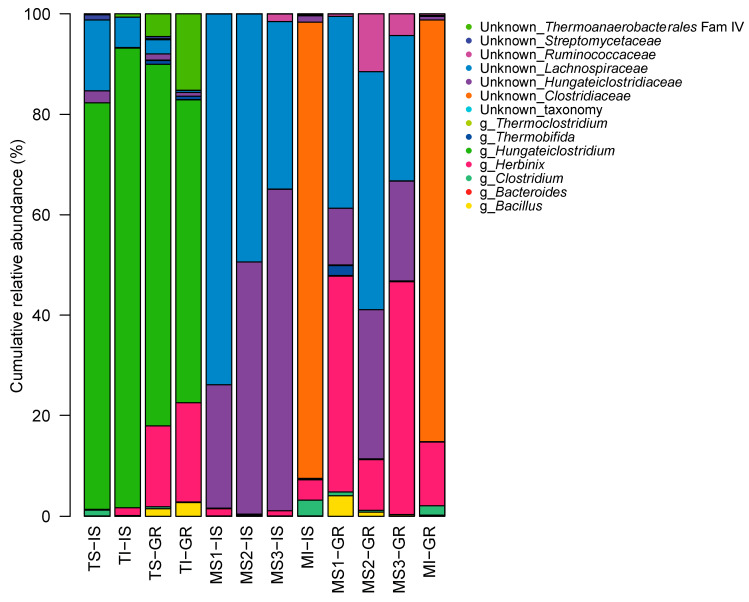
Taxonomic binning of GH48 gene sequences amplified in DNA of lab-scale biogas fermenters at genus level. TS—thermophilic stable; TI—thermophilic instable; MS—mesophilic stable; MI—mesophilic instable; IS—*in sacco*; GR—raw digestate.

Based on the GH48 classification outlined above, 17 different genera were identified on average in metagenomic DNA in all analyzed fermenter stages (Figure 3). Thereof, 50–99% belonged to unknown genera in mesophilic biogas fermentation. In contrast, only 7–18% of the GH48 gene sequences in the thermophilically operated biogas fermentation were unknown. In detail, metagenomic DNA of mesophilic fermenter samples MS1, MS2 and MS3 consisted in large proportions of GH48 gene sequences from unknown *Lachnospiraceae* (73.9%, 49.3% and 33.4% in IS and 38.1%, 47.4% and 29.0% in GR, respectively) and unknown *Hungateiclostridiaceae* (24.5%, 50.2% and 64.0% in IS and 11.3%, 29.7% and 20.0% in GR, respectively). Interestingly, the GH48 gene sequences in metagenomic DNA of instable, mesophilic fermenter stages (MI), either IS or GR, differed from the other samples, e.g., GH48 gene sequences of unknown *Clostridiaceae* were predominant (90.7% or 84.0%, respectively). In the thermophilic samples TS-IS, TI-IS, TS-GR and TI-GR, the GH48 gene sequences of *Hungateiclostridium* were most abundant (80.9%, 91.5%, 72.0% and 60.3%, respectively). Furthermore, GH48 gene sequences of unknown *Lachnospiraceae* were present in TS-IS (14.2%) and, in smaller parts, in TI-IS and TS-GR (6.0% and 2.9%, respectively), but were very low in TI-GR (0.4%). In contrast, GH48 gene sequences of *Herbinix* occurred in TS-GR and TI-GR (16.0% and 19.7%, respectively), less in TI-IS (1.5%) and very little in TS-IS (0.1%). A summary of the relative abundances for the different genera is given in Supplementary Material Table S9. A detailed analysis of the α–diversity based on the GH48 amplicons of all samples is summarized in Supplementary Material Table S10. The relative abundance of the individual OTUs in each sample, along with the accession number and corresponding identities [%] to the best hit in the NCBI database refseq_protein, identified via BLASTx, is given as Supplementary Material Table S11.

## 4. Discussion

With very few exceptions, the expression of GH48 module containing genes has been found to be a precondition of making bacteria truly cellulolytic [14,16,17]. This makes GH48 a valuable tool for identifying cellulolytic bacteria and to analyze their role in plant degrading ecosystems, a task that cannot be performed by using the 16S rDNA sequence alone. The novel primer mixture, consisting of 16 forward and 9 reverse primers, is a tool to quantify and identify cellulolytic bacteria via the amplification of GH48 gene sequences in DNA or RNA. Instead of using a single primer pair as performed by Pereyra et al., 2010 [14], the mixture is flexible in respect to expand the primer mixture with more individual primers for possibly novel evolving GH48 gene sequences in the future. In addition and in direct comparison to the primers of Pereyra et al., 2010 [14], a higher qPCR efficiency was achieved.

For the assignment of GH48 gene sequences to known organisms, a reliable reference database is necessary. Current GH48 gene containing databases comprise the dedicated CAZy database and general databases. However, despite CAZy.org [21] is a dedicated database on carbohydrate metabolic enzymes and documenting the protein name, organism name, protein accession number and experimental protein characterization data, this database lacks the nucleotide sequence allowing gene-based analyses. General databases like Uniprot (www.uniprot.org) and PDB (Protein Data Bank, www.rcsb.org) contain 375 entries from taxonomically classified bacteria [accessed 20-05-2019] and provide protein identification numbers and links to the gene for each GH48 enzyme. However, these databases also lack the ability to comprehensively search all GH48 genes. Thus, a comprehensive GH48 database having an emphasis on taxonomy was compiled in this study, allowing gene-based primer-finding and easy taxonomic classification. This novel database contains a taxonomic identifier (txid), the full taxonomic classification, the nucleotide sequences of GH48 and the 16S rRNA gene sequences of the corresponding organism (Supplementary Material File S1).

Similar to the 16S rRNA gene, in this work it is shown that the GH48 gene can be used as a molecular marker for genus (or even species) identification and OTU clustering using the full-length nucleotide sequence (about 2000 bp) or a highly conserved amplicon (about 344 bp). The choice of using the full-length sequence or the amplicon depends on the goal of the study. For species identification, use of the full-length GH48 nucleotide sequence is required as more identical amplicon sequences than identical full-length GH48 module nucleotide sequences were observed. On the other hand, identical full-length sequences were unlikely to be found in different species. However, modern sequencers for high-throughput (e.g., Illumina MiSeq, HiSeq 3000 and ThermoFisher Ion PGM) can only accurately sequence up to 150–400 bp per read [46], while the full-length gene sequences by far exceed this sequencing limit. Thus, for microbiota analysis, gene-specific amplicon sequencing is commonly used to assess microbial diversity. For biogas fermentation, the number and species of cellulolytic bacteria (i.e., based on GH48) are even more interesting than the number and species of bacterial organisms in general, based on 16S rRNA genes. However, a combination of both will be most informative.

GH48 thresholds for approximation of taxonomic boundaries were defined according to the corresponding gene sequence identities at certain 16S rRNA gene sequence identities using different subdatasets derived from the ‘Combined database’. Modeling the correlation of GH48 gene sequence to 16S rRNA gene sequence identities revealed that RMSE, MAE and PRESS of the polynomial model were lower than that of the linear model (Figure 1). As expected, both amplicon (Figure 1) and module sequence identities did not reach 100% at 100% 16S rRNA gene sequence identity, because some species (although rarely) contain more than one GH48 (Table 5). For instance, *Hungateiclostridium thermocellum* ATCC27405^T^ possesses two GH48: ABN53296.1 is a cellulosomal protein comprised of a GH48 module along with a dockerin type I domain (docI), whereas ABN51312.1 is a non-cellulosomal protein comprised of a GH48 module along with a carbohydrate binding module type 3 (CBM3). The GH48 modules of these two proteins share 54.48% identity on amino acid sequence level found using BLASTp. This also indicates that two proteins with redundant catalytic function, but distinct sites of action (i.e., soluble in the extracellular space or bound to the outer cell membrane assembled within a multi-enzyme protein complex like the cellulosome, respectively) can occur within one species.

Assuming that 97% 16S rRNA gene sequence identity correspond to the species boundary [47], sequencing reads can be classified into one species at 84% GH48 amplicon identity (Figure 1). To cross-validate this, another method that, surprisingly, needed 100% sequence identity to classify reads correctly was applied (Figure 2). However, excluding outliers, 94% sequence identity was needed. The disagreement between the two methods, calculating a distance-matrix or a clustering agreement, is substantial, but probably due to horizontal gene transfer of GH48 [48], such that species from different genera possess similar GH48 sequences (Supplementary Material Table S7). In fact, a high probability of GH48 horizontal gene transfer is observed among *Actinobacteria* and *Firmicutes* [49]. Consistently, most of the genera with similar GH48 amplicon sequence identity but low full-length 16S rRNA gene sequence identity belonged to *Actinobacteria* and *Firmicutes* indeed (Supplementary Material Table S8).

Clustering of GH48-gene amplicon sequencing reads at the proposed threshold of 94% identity enabled a comprehensive analysis of the cellulolytic microbiota at genus level. The advantage of combining the reads at genus level is a simplification of the comparison of the different fermenter stages tested, since the amplified sequence segment does not allow for identification at species level. In any case, in all samples, an average of 17 genera was identified indicating a very low diversity of cellulolytic organisms compared to the overall diversity of the microbial community in biogas fermenters [7]: e.g., 95 genera of Bacteria and Archaea in 21 full-scale biogas digesters [50] or 1641 bacterial 16S rRNA OTUs identified in 43 household biogas digesters [51].

Despite the low diversity of just 17 genera of cellulolytic genera in each experiment, a large number of these cellulolytic taxa are found to be novel, especially at mesophilic conditions, when using high-throughput sequencing of GH48 amplicons and the novel primer mix for amplification. The previous studies from Pereyra et al. 2010 and Izquierdo et al. 2010 compare either different feeding of sulfate-reducing bioreactors [14] or different thermophilic, cellulolytic enrichment cultures [16], respectively and used cloning of PCR products for gene sequencing instead of next-generation sequencing methods as presented in this study. Another more current study highlights that analyzing the genetic diversity, here using GH48 genes in soil [52], of different environmental samples can subsequently lead to the identification and characterization of novel enzymes, which may exhibit an improved function of interest.

The results obtained within this study clearly indicate that at mesophilic fermenter operation conditions most of the detected GH48 gene sequences cannot be assigned to a known organism. Thus, there is a high potential of identifying novel enzymes, indicating potentially new cellulolytic bacterial taxa. Hence, the results stress the urgent need of further isolation, characterization and genome sequencing of novel cellulolytic organisms. Detailed taxonomic identification of metagenomic data and subsequent characterization of enzymes of interest is only possible with a number of closely related reference species [53]. Only the recent characterization of organisms within the genus *Herbinix*, isolated in 2015 [54] and 2016 [55], enabled the assignment of amplicon reads to this genus in this study and also made the enzymatic characterization of the arabinoxylan-degrading enzymes of *H. hemicellulosilytica* possible, as described by Mechelke et al., 2017 [56]. Further, the recently isolated novel member of the genus *Hungateiclostridium*, *H. mesophilum* [57], confirms that there are still novel organisms within the cellulolytic biogas microbiota to be analyzed for gaining deeper insights into substrate degradation during the microbial biogas process.

Evaluating cellulolytic activity of hydrolytic bacteria at different fermenter stages using sequencing and absolute numbers of GH48 gene copies may be biased for each experimental condition due to differences in the quality of the extracted nucleic acids. Contamination of PCR templates, e.g., with humic acids, have a great impact on amplification efficiencies [58]. Therefore, the cellulolytic potential of a fermenter condition was defined as the ratio of bacterial GH48 gene copies to the total amount of bacterial 16S rRNA gene sequence copies (GH48/16S), quantified within the same nucleic acid sample. Admittedly, the ratio can be biased due to the different copy numbers of ribosomal RNA operons or possession of one or more GH48 genes in each strain. Interestingly, soil bacteria have an average of 1.4 or 5.5 copies of 16S rRNA genes in slow or fast growing organisms, respectively [15]. Consequently, the ‘true’ cellulolytic potential may be underestimated by a simple GH48 amplicon over 16S rRNA amplicon ratio. However, this ratio gives a hint into a relative cellulolytic potential at a certain time point and enables comparison of different samples or fermenter stages as shown in this study.

In general, higher amounts of GH48 genes in the metagenome, and in almost all samples of the metatranscriptome (except MS3), was observed in the *in sacco* enrichments (Table 4) compared to the GR samples of the same time point (Table 3). This was to be expected, since *in sacco* is a well-established method to enrich for cellulolytic organisms in their natural environment [19,30,31,59]. Nevertheless, to assess the cellulolytic potential of a certain biogas fermenter stage, the overall GH48 to 16S rRNA gene ratio in the digestate samples (GR) is more important as it represents the total bacterial community involved in the biogas process.

Interestingly, the observed values indicate higher ratios at the inefficient state MS1 as well as the instable state MI and TI compared to the stable and more efficient MS2, MS3 and TS (Table 3 and Table 4). Therefore, we conclude that the overall number of cellulolytic organisms increases with a higher volumetric load of the fermenters. Subsequently, this can lead to stress in the overall biogas process [60]. In contrast, the GH48-to-16S rRNA ratios in the metatranscriptome reflects the efficiency of the biogas fermenters: ratios are highly reduced in the inefficient states compared to the more efficient ones, although keeping in mind that quantification of GH48 gene copies is below a valid detection limit for some of the very inefficient fermenter states (Supplementary Material Table S6). These results indicate the importance of preferably analyzing the metatranscriptome instead or in addition to the metagenome to monitor the active community in a certain sample, but also reflect the emerging challenges, e.g., low extraction yields, while working with RNA samples.

## 5. Conclusions

The novel primer combination cel48-Mix2 can be applied efficiently for qPCR and RT-qPCR as well as for amplicon sequencing to quantify and identify cellulolytic bacteria in biogas fermenters and, presumably, also in other complex habitats. Since the novel primer mix covers a wide spectrum of GH48 gene sequences, a more accurate comparison of different environmental samples is possible. In addition, the novel database for GH48 genes facilitates the taxonomic identification of cellulolytic organisms via GH48 gene sequences. The proposed thresholds enable fast OTU clustering based on GH48 sequences, as well as direct taxonomic identification of novel metagenomic and metatranscriptomic sequencing data by comparison with the provided database. Moreover, sequencing of GH48 amplicons enabled identifying novel cellulolytic taxa, as was demonstrated in this study, in particular for biogas fermentations operated under mesophilic conditions. Therefore, for these samples, targeted isolation and characterization of the latter organisms may be important to elucidate the biogas microbiota in more detail. The novel primer mixture cel48-Mix2, applied to other environmental samples, will help identifying a greater variety of unknown cellulolytic organisms and, subsequently, aiding in their isolation.

## Figures and Tables

**Table 1:** Primer used for the amplification of target genes.

Primer-ID	Target Gene	Nucleotide Sequence	Source
Cel48_490F_I	GH48	T[I]ATGGTTGAAGCTCCDGAYTAYGG	Pereyra et al. 2010 [14], modified within this work
Cel48_920R_I	GH48	CCAAA[I]CCRTACCAGTTRTCAACRTC	
F919mod	bacterial 16S rRNA	GAATTGACGGGGRYCCGCACAAG	Rudi et al. 1997 [28] modified by Lebuhn et al. 2014 [12]
R1378mod	bacterial 16S rRNA	CGGTGTGTACAAGRCCCGRGAACG	Costa et al. 2006 [29], modified by Lebuhn et al. 2014 [12]
Cel48-Mix2F	GH48	Table 2	this work
Cel48-Mix2R	GH48	Table 2	

Degenerative positions following International Union of Pure and Applied Chemistry (IUPAC) nucleotide code: D—AGT; R—AG; Y—CT.

**Table 2:** Summary of primers to amplify glycoside hydrolases of family 48 (GH48) gene sequences.

ID	Sequence	Length (bp)	V (µL)	N (Degeneration)
F1	TCTTGAGTGAAGCTCCAGACTA	22	1	0
F2	TGCTTAGCGA[AG]GCGCCCAA	19	2	1
F3	GAGCGA[AG]GC[I]CC[I]GATTAC	19	2	1
F4	TGAGCGAGGCTCC[GT]GAC[CT]A	19	4	2
F5	TGATATGCGAAGCACCTGA[CT]TATG	24	2	1
F6	TCATATG[CT]GAAGC[GA]CC[TG]GATTAC	23	8	3
F7	TGATATGCGAGGCACCGGATTA	22	1	0
F8	TGAACTGTGAAGCTCCTGATCAA	23	1	0
F9	TGTGTGTTGAAGCGCCTGATTAC	23	1	0
F10	TGATTGT[I]GAAGCTCC[TG]GA[CT]TATG	24	4	2
F11	ATCGTCGAAGCGCCTGACCA	20	1	0
F12	ATCGACGAGGCGCCCGA	17	1	0
F13	TTTTGGTGGAAGCTCCGGACTAT	23	1	0
F14	TAATGGTTGAAGCACC[I]GAC[CT]AT	23	2	1
F15	TCATCGTCGA[GA]GC[I]CC[I]GA[CT]TA	22	4	2
F16n	ATCGTCGAGGC[I]CC[GC]GACC	19	2	1
R1	CCGAAGCCGTAGACGTTGTC	20	1	0
R2	CCGTAACCGTAGATGTTGTC[I]A	22	1	0
R3	CCG[AT]AGCCGTA[GC][GC]TGTTGTC	20	8	3
R4	CCGTATCCATACCAGTTATCCGTA	24	1	0
R5	CCAAATCCATACCAGTTATCACAGT	25	1	0
R6	CCAAAACCATACCAGTTATCAACATC	26	1	0
R7	CCATCCATACCAGTT[AG]TC[I]AC[AG]TC	24	4	2
R8	CCR[AT]A[I]CCGTAGACGTTGTC	20	4	1
R9	CCGAAGCCGTACCA[AG]TT[AG]TC	20	4	2

[]—degenerative base.

**Table 3:** GH48 and 16S rRNA gene copy numbers quantified in DNA isolated from raw digestate samples of biogas fermenters.

Sample	GH48 (Gene Copies/µL)	Standard Deviation	16S rRNA (Gene Copies/µL)	Standard Deviation	Ratio GH48/16S rRNA (%)
MS1	5.7 × 10^6^	±1.1 × 10^6^	7.1 × 10^8^	± 1.8 × 10^8^	0.8
MS2	4.8 × 10^6^	±3.1 × 10^5^	1.5 × 10^9^	± 3.6 × 10^8^	0.3
MS3	3.6 × 10^6^	±4.7 × 10^5^	1.2 × 10^9^	± 1.5 × 10^8^	0.3
MI	5.4 × 10^6^	±4.0 × 10^5^	6.7 × 10^8^	± 1.5 × 10^7^	0.8
TS	2.9 × 10^6^	±2.9 × 10^5^	8.0 × 10^8^	± 2.6 × 10^8^	0.4
TI	7.9 × 10^6^	±8.1 × 10^5^	1.2 × 10^9^	± 6.8 × 10^7^	0.7

Absolute gene copy numbers calculated from the standard curve measured within the same qPCR. Standard deviation calculated from three technical replicates.

**Table 4:** GH48 and 16S rRNA gene copy numbers quantified in DNA isolated from *in sacco* samples of biogas fermenters.

Sample	GH48 (Gene Copies/µL)	Standard Deviation	16S rRNA (Gene Copies/µL)	Standard Deviation	Ratio GH48/16S rRNA (%)
MS1	6.8 × 10^6^	±8.7 × 10^5^	2.2 × 10^8^	±5.1 × 10^7^	3.1
MS2	2.4 × 10^7^	±9.6 × 10^6^	6.4 × 10^8^	±1.1 × 10^7^	3.7
MS3	3.8 × 10^6^	±1.4 × 10^5^	1.9 × 10^8^	±2.0 × 10^7^	2.0
MI	9.1 × 10^4^	±1.3 × 10^4^	7.8 × 10^6^	±8.4 × 10^5^	1.2
TS	3.4 × 10^6^	±4.0 × 10^5^	2.4 × 10^8^	±4.1 × 10^7^	1.4
TI	5.8 × 10^6^	±1.5 × 10^6^	1.7 × 10^8^	±1.4 × 10^7^	3.5

Absolute gene copy numbers calculated from the standard curve measured within the same qPCR. Standard deviation calculated from three technical replicates.

**Table 5:** Parameters of the GH48 database.

Entries Summary		Sequence Length Distribution				
No. of entries	944			Min	Mean	Max
No. of genera	104	GH48 module containing protein (nucleotide)		1429	2926	7032
No. of families	42	GH48 module containing protein (amino acid)		475	975	2343
Entries with full-length 16S	580 (61%)	GH48 amplicon length (nucleotide, without primers)		291	344	379
Species or strains with >1 GH48	82 (8.7%)	16S rRNA gene sequence (nucleotide)		72	1459	1834
Entries with identical GH48 module	78 (8.3%)					
Entries with identical GH48 amplicon	184 (19.5%)					

## Data Availability

The NGS raw sequencing reads of the GH48 gene sequences in metagenomic DNA of the analyzed biogas fermenters are deposited at the Sequence Read Archive, NCBI, available under the accession numbers SRR11545268-79, BioProject no. PRJNA625239 and the corresponding BioSamples nos. SAMN14595678-89.

## Supplementary Materials

The following are available online at https://www.mdpi.com/2076-2607/8/9/1297/s1, Table S1: GH48 gene sequences used for the design of cel48-Mix2, Table S2: Lab-scale biogas fermenters, Table S3: BLASTx results to the NCBI refseq_protein database of GH48 gene sequences amplified via cel48_490F_I + cel48_920R_I [4] or cel48-Mix2, Table S4: Nucleic acid extraction from lab-scale fermenter operated at 38 °C, Table S5: Nucleic acid extraction from lab-scale fermenter operated at 55/52 °C, Table S6: GH48 and 16S rRNA gene copy numbers quantified in cDNA of biogas fermenters, Table S7: List of genera in the ‘Combined’ database, Table S8: Outliers in GH48-16S dataset, Table S9: Relative abundances [%] of GH48 gene sequences in the metagenome at different fermenter stages, summarized at genus level, Table S10: α-diversity of GH48 amplicon sequencing in metagenomic DNA, Table S11: Relative abundance of GH48 OTUs from metagenomic DNA of biogas fermenter samples in addition to the BLASTx result (highest score) in the NCBI refseq_protein database, Figure S1: Dissociation curve of PCR products amplified with cel48-Mix2 in the qPCR setup, Figure S2: Phylogenetic tree of GH48 gene sequences amplified from the metagenome of a mesophilically operated lab-scale biogas fermenter using cel48_490F-I+cel48_920R-I or cel48-Mix2 (highlighted in blue), Figure S3: Standard for the absolute quantification of GH48 gene sequences with linearized pET24c:*cel48S* and gDNA of *E. coli* as background, Figure S4: Amplicon length distribution, Script S1: GenPept2txid.py, Script S2: GenBank2Features.py, Script S3: GenPept216S.py. File S1: GH48_database.xlsx, File S2: GH48_modules.fasta.

## Abbreviations

CAZy(mes)	carbohydrate-active enzyme(s)
ddH2O	doubled deionized water
GH48	glycoside hydrolase of family 48
GR	digestate (from German: *Gärrest*)
IS	*in sacco*
MAE	mean absolute error
MI	mesophilic-instable
MPN	most probable number
MS	mesophilic-stable
NGS	next-generation sequencing
NTC	non-template control
OTU	operational taxonomic unit
PRESS	predicted residual error sum of squares
qPCR	quantitative polymerase chain reaction
RMSE	root-mean-square error
RT	reverse transcription
TI	thermophilic-instable
TS	thermophilic-stable
txid	taxonomic identification number
