# Supplementary material for: A Novel Primer Mixture for GH48 Genes: Quantification and Identification of Truly Cellulolytic Bacteria in Biogas Fermenters
Source: Microorganisms. 2020 Aug 25;8(9):1297. doi: 10.3390/microorganisms8091297 (PMC7565076; doi:10.3390/microorganisms8091297)
Supplement: Supplementary file 1 [file microorganisms-08-01297-s001.zip › Supplementary Material/Supplementary Material.pdf]

Supplementary Material

A novel primer mixture for GH48 genes improves quantification and identification of truly cellulolytic bacteria

Regina Rettenmaier [1], Yat Kei Lo [1], Larissa Schmidt [1], Bernhard Munk [2], Ilias Lagkouvardos [3], Klaus Neuhaus [3], Wolfgang Schwarz [4], Wolfgang Liebl [1] and Vladimir Zverlov [1,5]*

[1]Chair of Microbiology, Technical University of Munich, Emil-Ramann-Str. 4, 85354 Freising, Germany; regina.rettenmaier@tum.de; kei.lo@campus.lmu.de; schmidt_larissa@mail.ru; wliebl@wzw.tum.de

[2]Bavarian State Research Center for Agriculture, Central Department for Quality Assurance and Analytics, Lange Point 6, 85354 Freising, Germany; bernhard.munk@lfl.bayern.de

[3]ZIEL - Core Facility Microbiome, Technical University of Munich, Weihenstephaner Berg 3, 85354 Freising, Germany; neuhaus@tum.de; ilias.lagkouvardos@tum.de

[4]Aspratis GmbH. Munich, Germany, Hübnerstr. 11, 80637 Munich, Germany; schwarz@outlook.de [5]Institute of Molecular Genetics, RAS, Kurchatov Sq. 2, 123182 Moscow, Russia; vladimir.zverlov@tum.de

Correspondence author:

*Vladimir Zverlov, vladimir.zverlov@tum.de

Key words: amplicon sequencing; qPCR; biogas fermenters; taxonomy; DNA; RNA

Supplementary Material Table S1: GH48 gene sequences used for the design of cel48-Mix2.

Identification of genes was based on the CAZy database (Henrissat, 1991 [1]; version April 2017), the corresponding gene sequences were manually extracted from NCBI.

Accession	Organism	Accession	Organism
P000481.1	*Acidothermus cellulolyticus*	CP002360.1	*Mahella australiensis*
CP006272.1	*Actinoplanes friuliensis*	CP014275.1	*Martelella* sp.
AP012319.1	*Actinoplanes missouriensis*	CP002162.1	*Micromonospora aurantiaca*
CP003170.1	*Actinoplanes* sp.	CP002399.1	*Micromonospora* sp.
CP005929.1	*Actinoplanes* sp.	FJ544850.1	*Myxobacter* sp.
CP002000.1	*Amycolatopsis mediterranei*	CP002040.1	*Nocardiopsis dassonvillei*
CP016174.1	*Amycolatopsis orientalis*	CP000850.1	*Salinispora arenicola*
KP893101.1	*Bacillus licheniformis*	CP000667.1	*Salinispora tropica*
CP012110.1	*Bacillus licheniformis*	CP012382.1	*Streptomyces ambofaciens*
CP010524.1	*Bacillus paralicheniformis*	BA000030.4	*Streptomyces avermitilis*
CP000813.4	*Bacillus pumilus*	CP002047.1	*Streptomyces bingchenggensis*
CP009108.1	*Bacillus pumilus*	AL939128.1	*Streptomyces coelicolor*
AY374129.1	*Bacteroides cellulosolvens*	CP006259.1	*Streptomyces collinus*
CP001393.1	*Caldicellulosiruptor bescii*	CP010849.1	*Streptomyces cyaneogriseus*
CP002330.1	*Caldicellulosiruptor kronotskyensis*	HE971709.1	*Streptomyces davawensis*
CP003001.1	*Caldicellulosiruptor lactoaceticus*	CP009438.1	*Streptomyces glaucescens*
CP002164.1	*Caldicellulosiruptor obsidiansis*	CP003275.1	*Streptomyces hygroscopicus*
L32742.1	*Caldocellum saccharolyticum*	LK022848.1	*Streptomyces iranensis*
CP001700.1	*Catenulispora acidiphila*	LN831790.1	*Streptomyces leeuwenhoekii*
L38827.1	*Cellulomonas fimi*	CP009124.1	*Streptomyces lividans*
CP001964.1	*Cellulomonas flavigena*	CP009313.1	*Streptomyces nodosus*
AEI13168.1	*Cellulomonas gilvus*	CP002475.1	*Streptomyces pratensis*
GU211268.1	*Cellulosilyticum ruminicola*	CP011340.1	*Streptomyces pristinaespiralis*
AE001437.1	*Clostridium acetobutylicum*	CP006567.1	*Streptomyces rapamycinicus*
HG917869.1	*Clostridium bornimense*	LN997842.1	*Streptomyces reticuli*
CP001348.1	*Clostridium cellulolyticum*	FN554889.1	*Streptomyces scabiei*
LM995447.1	*Clostridium cellulosi*	CP011799.1	*Streptomyces* sp.
U34793.3	*Clostridium cellulovorans*	CP011492.1	*Streptomyces* sp.
CP003065.1	*Clostridium clariflavum*	CP013743.1	*Streptomyces* sp.
AB004845.1	*Clostridium josui*	CP009754.1	*Streptomyces* sp.
CP002582.1	*Clostridium lentocellum*	CP013142.1	*Streptomyces* sp.
CP000885.1	*Clostridium phytofermentans*	CP002993.1	*Streptomyces* sp.
CP004121.1	*Clostridium saccharoperbutylacetonicum*	CP015098.1	*Streptomyces* sp.
CP014673.1	*Clostridium stercorarium*	CP003990.1	*Streptomyces* sp.
GQ487568.1	*Clostridium straminisolvens*	FR845719.1	*Streptomyces venezuelae*
CP000568.1	*Clostridium thermocellum*	CP013129.1	*Streptomyces venezuelae*

CP013828.1	*Ruminiclostridium thermocellum*	CP010407.1	*Streptomyces vietnamensis*
AJ863163.1	*Ruminiclostridium thermocellum*	CP009922.2	*Streptomyces xiamenensis*
CP003259.1	*Clostridium* sp.	CP001814.1	*Streptosporangium roseum*
CP000155.1	*Hahella chejuensis*	AF144563.1	*Thermobifida fusca*
LN879430.1	*Herbinix* sp.	CP001874.1	*Thermobispora bispora*
CP000875.1	*Herpetosiphon aurantiacus*	CP002638.1	*Verrucosispora maris*
CP001706.1	*Jonesia denitrificans*	CP001821.1	*Xylanimonas cellulosilytica*
AP010968.1	*Kitasatospora sp.*		

1. Henrissat, B. A classification of glycosyl hydrolases based on amino acid sequence similarities. Biochem. J. 1991, 280 (Pt 2), 309–316.

Supplementary Material Table S2: Lab-scale biogas fermenters.

fermenter stage ID	temperature [°C]	date of sample	volumetric load of fermenter [kg_{VS}/m^3*d] at date of sampling	Start of *in sacco* incubation	volumetric load of fermenter [kg_{VS}/m^3*d] at date of incubation start
MS1	38	13.03.19	2.75	08.03.2019	2.00
MS2	38	13.05.19	4.00	08.05.2019	4.00
MS3	38	12.07.19	5.50	07.07.2019	5.50
MI	38	07.08.19	0.00	02.08.2019	0.00
TS	55	02.05.19	1.25	27.04.2019	1.25
TI	52	12.07.19	18.00	07.07.2019	4.00

M, mesophilic; T, thermophilic; S, stable, I, instable; VS, volatile solids

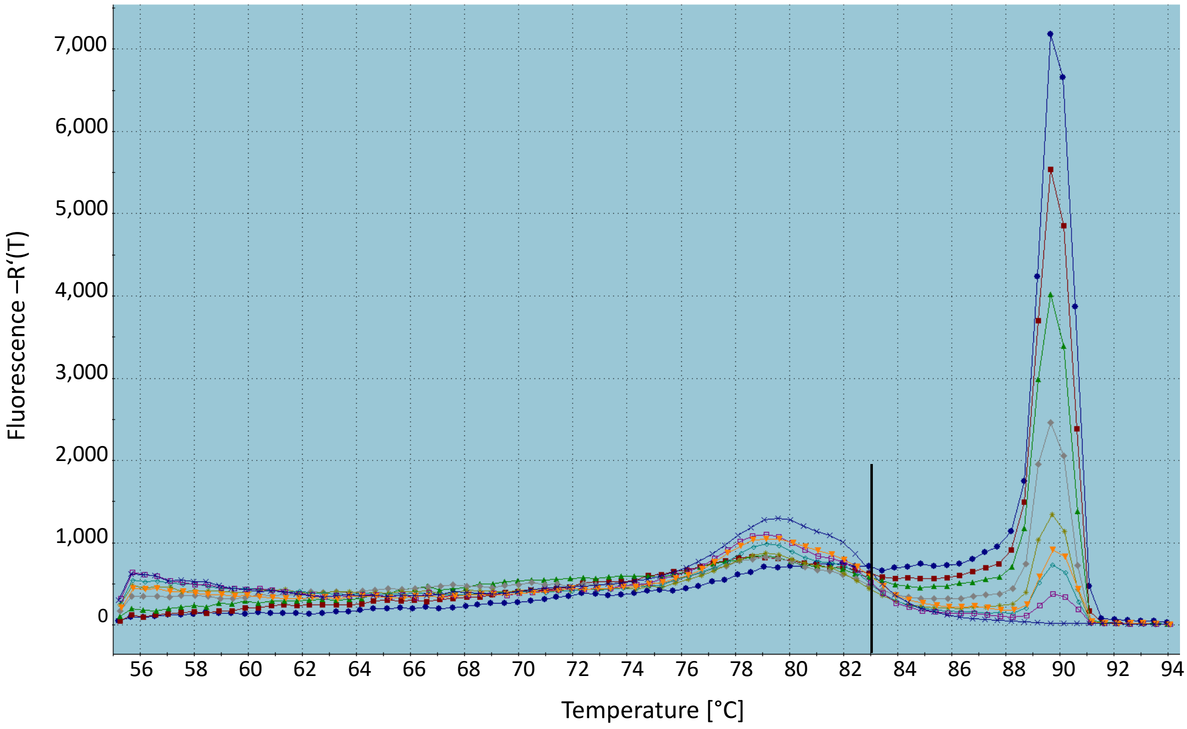

Supplementary Material Figure S1: Dissociation curve of PCR products amplified with cel48-Mix2 in the qPCR setup.

The specific PCR product amplified of pET24c:*cel48A*, linearized plasmid DNA, dissociated at an average temperature of 90 °C and was not detected in the non-template control (NTC). Primer-dimer formation was highest in the NTC, but was observed in all samples. Dissociation of these primer dimers was observed in-between approximately 76-83 °C. To avoid the quantification of these primer dimers, a dissociation step for 10 s at 83 °C was inserted before quantification after each PCR cycle. Legend: 10^2-10^9 GH48 gene copies/µl blue; red; green; grey; yellow; cyan; purple, respectively; blue: NTC.

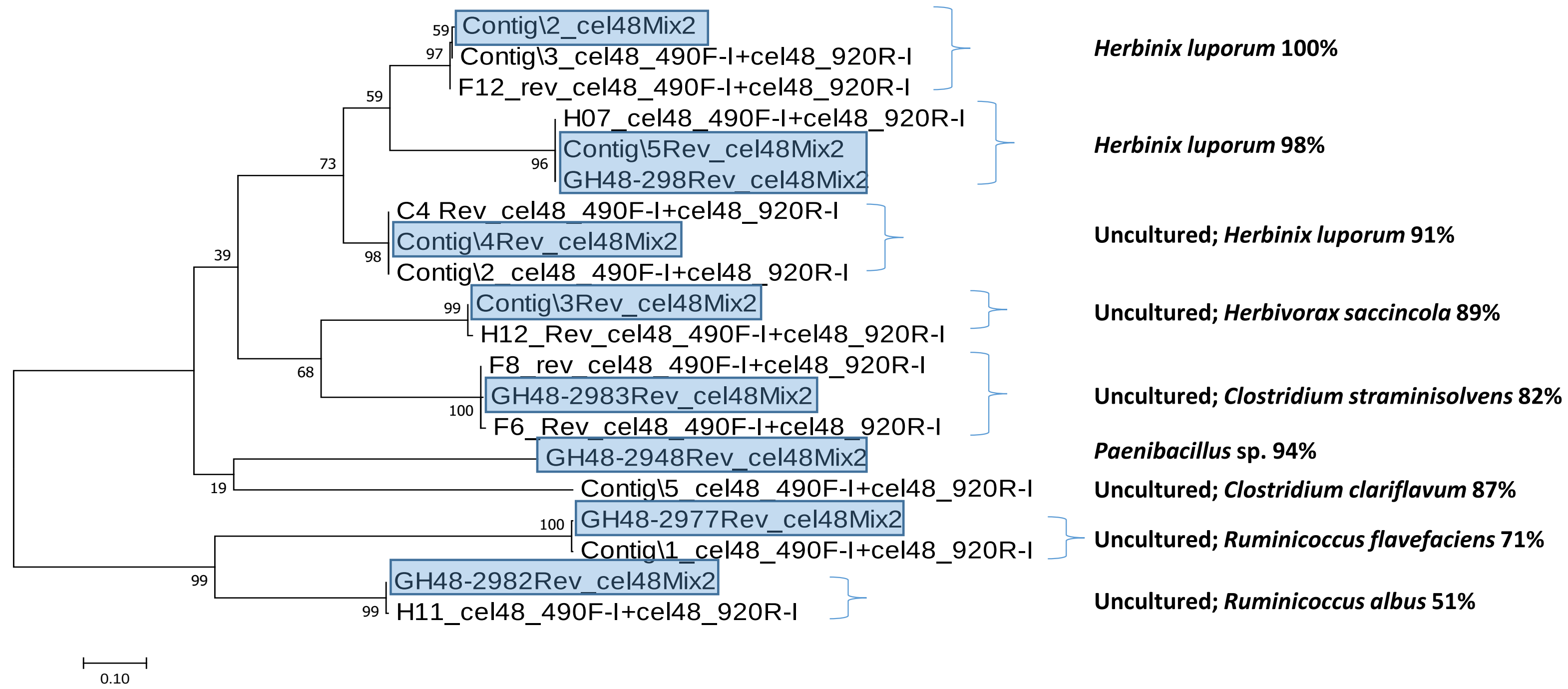

Supplementary Material Figure S2: Phylogenetic tree of GH48 gene sequences amplified from the metagenome of a mesophilically operated lab-scale biogas fermenter using cel48_490F-I+cel48_920R-I or cel48-Mix2 (highlighted in blue). Identical GH48 gene sequences were combined as contigs. Alignment was performed with MEGA7 [2] applying the ClustalW algorithm. The tree was constructed via the Maximum Likelihood method based on the Tamura-Nei model [3] with 1,000 bootstraps. Bootstrap values (>50) are shown at branch nodes. Sequence identities in [%] to the next organisms are based on comparison to the NCBI database for non-redundant protein sequences by means of Blastx.

2. Kumar, S.; Stecher, G.; Tamura, K. MEGA7: Molecular Evolutionary Genetics Analysis Version 7.0 for Bigger Datasets. Mol. Biol. Evol. 2016, 33, 1870–1874, doi:10.1093/molbev/msw054.

3. Tamura, K.; Nei, M. Estimation of the number of nucleotide substitutions in the control region of mitochondrial DNA in humans and chimpanzees. Mol. Biol. Evol. 1993, 10, 512–526, doi:10.1093/oxfordjournals.molbev.a040023.

Supplementary Material Table S3: Blastx results to the NCBI refseq_protein database of GH48 gene sequences amplified via cel48_490F_I + cel48_920R_I [4] or cel48-Mix2.

Sequence	Primer	Blastx Result (NCBI refseq_protein)	
		Identity [%]	Accesion
Contig\1	cel48_490F_I & cel48_920R_I	74.4	WP_173332627.1
Contig\2		89.4	WP_058258585.1
Contig\3		98.4	WP_058258585.1
C4 Rev		89.4	WP_058258585.1
Contig\5		86.7	WP_014256942.1
H12_Rev		86.3	WP_105367686.1
H07		98.4	WP_058258121.1
H11		55.4	WP_072416445.1
F6_Rev		81.7	WP_128705948.1
F8_rev		81.7	WP_128705948.1
F12_rev		97.6	WP_058258585.1
Contig\2	cel48-Mix2	100.0	WP_058258585.1
Contig\3Rev		89.3	WP_105367686.1
Contig\4Rev		90.5	WP_058258585.1
GH48_2977Rev		74.1	WP_173549419.1
GH48_298Rev		57.9	WP_177531663.1
GH48_2983Rev		78.5	WP_128705948.1
GH48_2948Rev		93.9	WP_036690606.1
Contig\5Rev		100.0	WP_058258121.1
GH48_2982Rev		93.3	WP_058258121.1

4. Pereyra, L.P.; Hiibel, S.R.; Prieto Riquelme, M.V.; Reardon, K.F.; Pruden, A. Detection and quantification of functional genes of cellulose-degrading, fermentative, and sulfate-reducing bacteria and methanogenic archaea. Appl. Environ. Microbiol. 2010, 76, 2192–2202, doi:10.1128/AEM.01285-09.

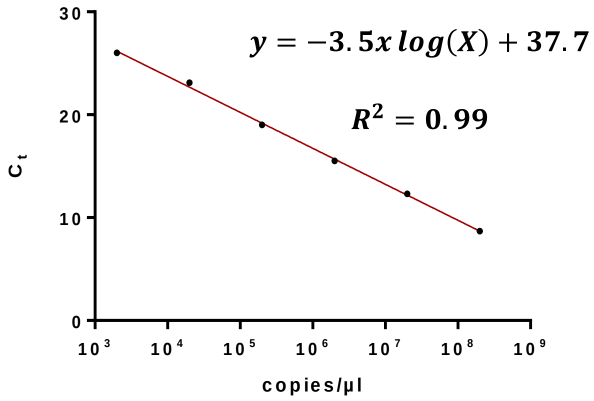

Supplementary Material Figure S3: Standard for the absolute quantification of GH48 gene sequences with linearized pET24c:*cel48S* and gDNA of *E. coli* as background.

Supplementary Material Table S4: Nucleic acid extraction from lab-scale fermenter operated at 38 °C.

		DNA			RNA		
	Sample	[ng/µl]	A260	A260/A280	[ng/µl]	A260	A260/A280
MS1	IS I	16.35	0.031	1.82	24.31	0.061	2.00
	IS II	32.10	0.072	1.86	16.01	0.040	2.27
	IS III	81.66	0.164	1.68	27.43	0.069	2.17
	GR I	220.96	0.431	1.81	91.50	0.229	1.83
	GR II	346.34	0.712	1.73	93.09	0.233	1.76
	GR III	336.89	1.188	1.62	89.03	0.223	1.85
MS2	IS I	121.90	0.250	1.89	41.63	0.104	1.85
	IS II	138.10	0.279	2.00	34.28	0.086	1.76
	IS III	162.60	0.332	1.99	9.33	0.023	1.34
	GR I	390.60	0.810	1.98	53.55	0.134	1.73
	GR II	326.50	0.694	1.97	69.11	0.173	1.66
	GR III	299.70	0.630	1.98	62.19	0.155	1.83
MS3	IS I	73.10	0.148	1.97	45.42	0.114	1.80
	IS II	70.50	0.142	1.98	32.58	0.081	1.93
	IS III	75.30	0.154	1.97	69.01	0.173	1.55
	GR I	357.60	0.749	1.95	213.80	0.534	2.09
	GR II	364.2	0.749	1.97	207.57	0.519	2.11
	GR III	450.4	0.939	1.95	203.61	0.509	2.02
MI	IS I	7.10	0.021	1.81	21.53	0.054	1.28
	IS II	5.90	0.021	2.03	22.08	0.055	1.21
	IS III	8.10	0.026	1.95	16.49	0.041	1.27
	GR I	232.10	0.485	1.97	70.79	0.177	2.10
	GR II	169.80	0.349	1.98	54.73	0.137	1.95
	GR III	228.90	0.467	1.97	75.14	0.188	2.22

MS, mesophilic stable; MI, mesophilic instable; GR, digestate; IS, *in sacco*

I, II III, technical replicates

Supplementary Material Table S5: Nucleic acid extraction from lab-scale fermenter operated at 55/52 °C.

		DNA			RNA		
	Sample	c [ng/µl]	A260	A260/A280	c [ng/µl]	A260	A260/A280
TS	IS I	174.40	0.358	2.03	37.95	0.095	2.07
	IS II	130.70	0.265	2.04	36.06	0.09	1.8
	IS III	155.20	0.312	2.08	72.86	0.182	1.5
	GR I	248.90	0.529	2.00	21.70	0.054	1.63
	GR II	219.90	0.492	1.98	134.73	0.337	1.15
	GR III	201.70	0.435	1.95	19.59	0.049	1.54
TI	IS I	104.90	0.253	1.97	49.97	0.125	1.62
	IS II	122.60	0.522	2.05	56.32	0.141	2.09
	IS III	129.50	0.288	1.97	46.90	0.117	1.74
	GR I	307.30	0.660	1.97	210.95	0.527	2.18
	GR II	276.10	0.595	1.97	304.50	0.761	2.16
	GR III	378.60	0.788	1.95	349.16	0.873	2.11

TS, thermophilic stable; TI, thermophilic instable; GR, digestate; IS, *in sacco*

I, II III, technical replicates

Supplementary Material Table S6: GH48 and 16S rRNA gene copy numbers quantified in cDNA of biogas fermenters.

Sample	GH48 [gene copies/µl]	Standard deviation	16S rRNA [gene copies/µl]	Standard deviation	Ratio GH48 / 16S rRNA [%]
MS1-IS	8.5E+04	9.2E+03	1.0E+09	8.4E+07	0.008
MS1-GR	n.a.	n.a.	4.2E+07	5.4E+06	n.a.
MS2-IS	4.4E+04	3.6E+03	1.1E+09	1.7E+08	0.004
MS2-GR	n.a.	n.a.	1.1E+08	3.6E+07	n.a.
MS3-IS	5.8E+03	5.4E+02	9.8E+08	3.2E+07	0.0006
MS3-GR	1.0E+05	3.8E+03	3.4E+08	6.7E+07	0.03
MI-IS	n.a.	n.a.	9.1E+07	2.8E+06	n.a.
MI-GR	4.39E+04	3.3E+03	2.0E+09	9.3E+07	0.002
TS-IS	4.4E+04	4.0E+03	5.4E+08	4.2E+07	0.008
TS-GR	n.a.	n.a.	7.6E+07	6.2E+06	n.a.
TI-IS	3.7E+05	7.1E+03	1.8E+09	2.3E+08	0.021
TI-GR	2.7E+04	2.4E+03	9.9E+08	4.1E+07	0.003

MS, mesophilic stable; MI, mesophilic instable; TS, thermophilic stable; TI, thermophilic instable; GR, digestate; IS, *in sacco*; n.a., not available

Supplementary Material Table S7: List of genera in the 'Combined' database.

Genus	Count	Genus	Count	Genus	Count
Acidothermus	1	*Gorillibacterium*	2	*Paenibacillus*	93
Actinoalloteichus	7	*Hahella*	2	*Persicobacter*	1
Actinobacteria	5	*Herbidospora*	12	*Phytoactinopolyspora*	1
Actinomadura	7	*Herbinix*	3	*Plantactinospora*	4
Actinophytocola	1	*Herpetosiphon*	3	*Polyangium*	1
Actinoplanes	16	*Hungateiclostridium*	14	*Prauserella*	1
Actinospica	1	*Isoptericola*	2	*Promicromonospora*	3
Actinosynnema	3	*Janthinobacterium*	1	*Pseudosporangium*	1
Allonocardiopsis	1	*Jishengella*	3	*Reichenbachiella*	1
Amycolatopsis	16	*Jonesia*	2	*Rhizobacter*	1
Anaerobacterium	1	*Kibdelosporangium*	1	*Roseateles*	2
Aquimarina	12	*Kitasatospora*	12	*Ruminiclostridium*	2
Ardenticatena	1	*Kribbella*	1	*Ruminococcaceae*	1
Asanoa	3	*Kutzneria*	2	*Ruminococcus*	3
Azotobacter	1	*Labilibacter*	1	*Saccharothrix*	8
Bacillus	38	*Labilibaculum*	1	*Salinispora*	4
Bacteroides	2	*Lachnoclostridium*	2	*Sanguibacter*	1
Caldicellulosiruptor	18	*Lachnospiraceae*	1	*Sphaerisporangium*	1
Catellatospora	2	*Lachnotalea*	1	*Streptacidiphilus*	6
Catenulispora	1	*Lechevalieria*	6	*Streptomonospora*	1
Catenuloplanes	1	*Lentzea*	11	*Streptomyces*	382
Cellulomonas	16	*Mahella*	1	*Streptosporangium*	6
Cellulosimicrobium	2	*Marinateneraspora*	3	*Tenacibaculum*	2
Cellvibrionaceae	1	*Martelella*	1	*Thermoactinospora*	1
Clostridium	27	*Massilia*	1	*Thermobifida*	4

Cohnella	1	*Methylibium*	1	*Thermobispora*	1
Crossiella	1	*Microbispora*	4	*Thermoclostridium*	4
Cystobacter	1	*Micromonospora*	74	*Thermogemmatispora*	3
Dactylosporangium	1	*Micromonosporaceae*	2	*Thermosporothrix*	1
Flammeovirga	5	*Minicystis*	1	*Thermostaphylospora*	1
Flavivirga	1	*Myceligenerans*	1	*Umezawaea*	1
Fontibacillus	1	*Nocardia*	1	*Verrucosispora*	5
Geofilum	1	*Nocardiopsis*	9	*Vitiosangium*	1
Glycomyces	7	*Nonomuraea*	8	*Xiangella*	1
				Xylanimonas	1

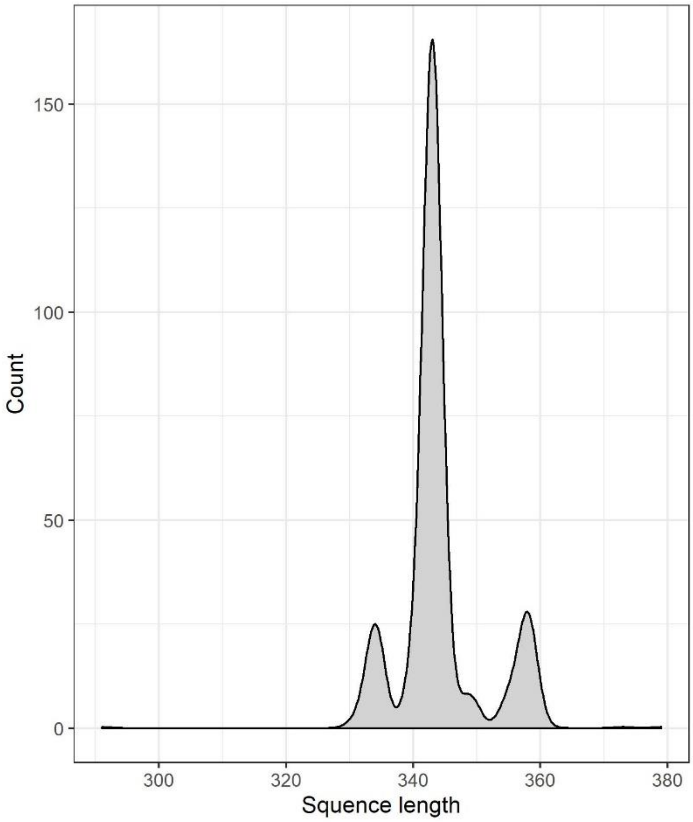

Supplementary Material Figure S4: Amplicon length distribution.

Supplementary Material Table S8: Outliers in GH48-16S dataset.

Genera	Abundance	GH48 amplicon identity [%]	16S rRNA identity [%]
Streptomyces - Streptomyces	355 (82.2%)	84.3 – 96.8	75.8 – 84.5
Jishengella - Jishengella	2 (0.5%)	89.5 – 89.8	76.8 – 76.9
Thermobifida - Thermobifida	2 (0.5%)	88.6 – 88.9	81.1
Glycomyces - Glycomyces	1 (0.2%)	94.0	78.5
Jishengella - Micromonospora	43 (10%)	84.7 – 89.8	76.0 – 76.9
Actinobacteria - Streptomyces	12 (2.8%)	84.8 – 89.5	76.8 – 84.3
Jishengella - Verrucosispora	5 (1.2%)	88.6 – 93.0	76.7 – 76.9
Jishengella - Salinispora	2 (0.5%)	84.7 – 85.0	76.7

Supplementary Material Table S9: Relative abundances [%] of GH48 gene sequences in the metagenome at different fermenter stages, summarized at genus level.

	**TS-IS**	**TI-IS**	**TS-GR**	**TI-GR**	**MS1-IS**	**MS2-IS**	**MS3-IS**	**MI-IS**	**MS1-GR**	**MS2-GR**	**MS3-GR**	**MI-GR**
g_*Bacillus*	0.06	0.10	1.48	2.73	0.01	0.00	0.02	0.06	4.09	0.77	0.24	0.19
g_*Bacteroides*	0.00	0.00	0.00	0.00	0.00	0.12	0.00	0.00	0.00	0.00	0.00	0.00
g_*Clostridium*	1.15	0.04	0.43	0.12	0.04	0.00	0.00	3.10	0.71	0.34	0.10	1.89
g_*Herbinix*	0.11	1.52	16.01	19.72	1.43	0.15	1.03	4.11	42.95	10.16	46.33	12.66
g_*Hungateiclostridium*	80.94	91.48	72.02	60.33	0.03	0.12	0.00	0.04	0.17	0.09	0.05	0.01
g_*Thermobifida*	0.01	0.04	0.79	0.66	0.01	0.00	0.01	0.02	1.98	0.04	0.03	0.03
g_*Thermoclostridium*	0.00	0.01	0.00	0.00	0.01	0.00	0.00	0.00	0.05	0.00	0.01	0.00
Unknown_taxonomy	0.00	0.00	0.03	0.00	0.00	0.00	0.00	0.18	0.00	0.00	0.00	0.02
Unknown_*Clostridiaceae*	0.00	0.00	0.01	0.00	0.10	0.00	0.01	90.79	0.04	0.00	0.04	84.04
Unknown_*Hungateiclostridiaceae*	2.37	0.13	1.25	0.77	24.50	50.24	64.01	1.29	11.33	29.70	19.96	0.67
Unknown_*Lachnospiraceae*	14.16	6.02	2.88	0.39	73.85	49.30	33.35	0.22	38.14	47.36	28.97	0.18
Unknown_*Ruminococcaceae*	0.00	0.00	0.15	0.01	0.02	0.06	1.55	0.18	0.53	11.53	4.29	0.33
Unknown_*Streptomycetaceae*	1.06	0.00	0.42	0.04	0.00	0.00	0.00	0.00	0.02	0.00	0.00	0.00
Unknown_*Thermoanaerobacterales* Fam. IV	0.15	0.67	4.54	15.23	0.00	0.00	0.01	0.00	0.00	0.00	0.00	0.00
Sum of unknown genera	17.73	6.82	9.28	16.44	98.48	99.60	98.94	92.66	50.05	88.60	53.25	85.23
Sum of known genera	82.27	93.18	90.72	83.56	1.52	0.40	1.06	7.34	49.95	11.40	46.75	14.77
Sum	100	100	100	100	100	100	100	100	100	100	100	100

MS, mesophilic stable; MI, mesophilic instable; TS, thermophilic stable; TI, thermophilic instable; GR, digestate; IS, *in sacco*

Supplementary Material Table S10: α-diversity of GH48 amplicon sequencing in metagenomic DNA.

Sample	*Richness*	*Evenness*
MS1-IS	13	0.414
MS1-GR	19	0.430
MS2-IS	16	0.238
MS2-GR	18	0.421
MS3-IS	11	0.354
MS3-GR	17	0.429
MI-IS	22	0.355
MI-GR	17	0.397
TS-IS	19	0.195
TS-GR	21	0.258
TI-IS	13	0.107
TI-GR	14	0.327

MS, mesophilic stable; MI, mesophilic instable; TS, thermophilic stable; TI, thermophilic instable; GR, digestate; IS, *in sacco*

Supplementary Material Table S11: Relative abundance of GH48 OTUs from metagenomic DNA of biogas fermenter samples in addition to the Blastx result (highest score) in the NCBI refseq_protein database.

OTU_Id	Biogas Fermenter Sample												Taxonomy	Blastx Result (NCBI refseq_protein)	
	MS1-IS	MS1-GR	MS2-IS	MS2-GR	MS3-IS	MS3-GR	MI-IS	MI-GR	TS-IS	TI-GR	TI-IS	TS-GR		Identity [%]	Accesion
OTU_8	44.7	33.1	49.1	45.7	33.0	27.3	0.1	0.1	1.6	0.0	0.1	1.8	*Bacteria;Firmicutes;Clostridia;Clostridiales;Lachnospiraceae;;;*	90.5	WP_058258585.1
OTU_15	24.8	4.1	0.1	1.4	0.3	1.4	0.1	0.0	0.5	0.0	0.0	0.4	*Bacteria;Firmicutes;Clostridia;Clostridiales;Lachnospiraceae;;;*	84.5	WP_058258121.1
OTU_11	14.8	6.1	45.6	17.4	50.0	14.6	0.1	0.4	0.6	0.0	0.0	0.3	*Bacteria;Firmicutes;Clostridia;Clostridiales;Hungateiclostridiaceae;;;*	80.7	WP_128705948.1
OTU_13	6.0	1.3	1.8	2.4	11.7	2.1	0.4	0.1	0.3	0.0	0.0	0.6	*Bacteria;Firmicutes;Clostridia;Clostridiales;Hungateiclostridiaceae;;;*	85.0	WP_105367686.1
OTU_41	4.3	0.9	0.0	0.3	0.0	0.3	0.0	0.0	0.2	0.0	0.0	0.2	*Bacteria;Firmicutes;Clostridia;Clostridiales;Lachnospiraceae;;;*	76.7	WP_004623851.1
OTU_3	2.3	2.7	1.7	6.5	1.2	1.4	0.5	0.1	0.1	0.0	0.0	0.1	*Bacteria;Firmicutes;Clostridia;Clostridiales;Hungateiclostridiaceae;;;*	86.7	WP_014256942.1
OTU_28	1.2	1.2	0.8	3.3	1.1	1.8	0.0	0.0	0.1	0.5	0.0	0.2	*Bacteria;Firmicutes;Clostridia;Clostridiales;Hungateiclostridiaceae;;;*	88.4	WP_105367686.1
OTU_18	1.1	31.4	0.1	7.3	0.8	33.8	3.0	9.5	0.1	14.0	1.1	12.2	*Bacteria;Firmicutes;Clostridia;Clostridiales;Lachnospiraceae;Herbinix;;*	78.5	WP_084124186.1
OTU_21	0.3	11.5	0.0	2.9	0.3	12.5	1.1	3.2	0.0	5.7	0.4	3.8	*Bacteria;Firmicutes;Clostridia;Clostridiales;Lachnospiraceae;Herbinix;;*	100.0	WP_058258121.1
OTU_17	0.2	0.0	0.2	0.0	0.0	0.0	0.0	0.0	0.0	0.0	0.0	0.0	*Bacteria;Firmicutes;Clostridia;Clostridiales;Hungateiclostridiaceae;;;*	77.2	WP_128705948.1
OTU_24	0.1	0.0	0.0	0.0	0.0	0.0	0.0	0.0	0.0	0.0	0.0	0.0	*Bacteria;Firmicutes;Clostridia;Clostridiales;Clostridiaceae;;;*	80.7	WP_081927072.1
OTU_43	0.0	0.0	0.0	0.0	0.0	0.0	0.0	0.0	0.0	0.0	0.0	0.0	*Bacteria;Firmicutes;Clostridia;Clostridiales;Clostridiaceae;;;*	82.5	WP_081927072.1
OTU_1	0.0	0.7	0.0	0.3	0.0	0.1	0.0	0.0	1.2	0.1	0.0	0.4	*Bacteria;Firmicutes;Clostridia;Clostridiales;Clostridiaceae;Clostridium;;*	100.0	WP_052659889.1
OTU_44	0.0	0.5	0.0	11.1	1.1	2.6	0.0	0.0	0.0	0.0	0.0	0.2	*Bacteria;Firmicutes;Clostridia;Clostridiales;Ruminococcaceae;;;*	66.9	WP_177531663.1
OTU_29	0.0	4.1	0.0	0.8	0.0	0.2	0.1	0.2	0.1	2.7	0.1	1.5	*Bacteria;Firmicutes;Bacilli;Bacillales;Bacillaceae;Bacillus;;*	100.0	WP_061578506.1
OTU_52	0.0	0.0	0.0	0.0	0.0	0.0	0.0	0.0	0.0	0.0	0.0	0.0	*Bacteria;Firmicutes;Clostridia;Clostridiales;Hungateiclostridiaceae;;;*	65.2	WP_105367686.1
OTU_5	0.0	0.0	0.1	0.0	0.0	0.0	0.0	0.0	80.0	60.3	91.3	71.9	*Bacteria;Firmicutes;Clostridia;Clostridiales;Hungateiclostridiaceae;Hungateiclostridium;;*	99.1	WP_105367686.1
OTU_10	0.0	0.0	0.0	0.0	0.0	0.0	0.0	0.0	0.0	0.0	0.0	0.0	*Bacteria;Firmicutes;Clostridia;Clostridiales;Hungateiclostridiaceae;;;*	100.0	WP_128705948.1
OTU_2	0.0	0.1	0.0	0.0	0.0	0.0	0.0	0.0	0.7	0.0	0.0	0.1	*Bacteria;Firmicutes;Clostridia;Clostridiales;Hungateiclostridiaceae;Hungateiclostridium;;*	100.0	WP_003516749.1

OTU_38	0.0	2.0	0.0	0.0	0.0	0.0	0.0	0.0	0.0	0.7	0.0	0.8	*Bacteria;Actinobacteria;Actinobacteria;Streptosporangiales;Nocardiopsaceae;Thermobifida;;*	100.0	WP_016188914.1
OTU_22	0.0	0.1	0.0	0.0	0.0	0.0	0.0	0.0	0.0	0.0	0.0	0.0	*Bacteria;Firmicutes;Clostridia;Clostridiales;Hungateiclostridiaceae;Thermoclostridium;;*	100.0	WP_015359554.1
OTU_74	0.0	0.0	0.0	0.0	0.0	0.0	0.3	0.0	1.1	0.2	0.0	0.1	*Bacteria;Firmicutes;Clostridia;Clostridiales;Hungateiclostridiaceae;;;*	100.0	WP_004623851.1
OTU_20	0.0	0.0	0.0	0.0	0.0	0.0	0.0	0.0	0.0	0.0	0.0	0.0	*Bacteria;Firmicutes;Clostridia;Clostridiales;Hungateiclostridiaceae;;;*	91.4	WP_015359554.1
OTU_27	0.0	0.0	0.0	0.0	0.0	0.0	0.0	0.0	0.1	15.2	0.7	4.5	*Bacteria;Firmicutes;Clostridia;Thermoanaerobacterales;Thermoanaerobacterales Family IV;;;*	79.1	WP_027621856.1
OTU_66	0.0	0.0	0.0	0.0	0.0	0.0	0.0	0.0	1.1	0.0	0.0	0.4	*Bacteria;Actinobacteria;Actinobacteria;Streptomycetales;Streptomycetaceae;;;*	77.2	WP_015771418.1
OTU_31	0.0	0.0	0.0	0.0	0.0	0.0	0.0	0.0	11.9	0.4	6.0	0.5	*Bacteria;Firmicutes;Clostridia;Clostridiales;Lachnospiraceae;;;*	69.5	WP_127352229.1
OTU_113	0.0	0.0	0.0	0.0	0.0	0.0	3.1	1.9	0.0	0.0	0.0	0.0	*Bacteria;Firmicutes;Clostridia;Clostridiales;Clostridiaceae;Clostridium;;;*	76.1	WP_084124186.1
OTU_26	0.0	0.0	0.0	0.0	0.0	0.0	26.1	23.1	0.0	0.0	0.0	0.0	*Bacteria;Firmicutes;Clostridia;Clostridiales;Clostridiaceae;;;*	75.0	WP_084124186.1
OTU_482	0.0	0.0	0.0	0.0	0.0	0.0	1.8	1.7	0.0	0.0	0.0	0.0	*Bacteria;Firmicutes;Clostridia;Clostridiales;Clostridiaceae;;;*	72.2	WP_051629492.1
OTU_49	0.0	0.0	0.0	0.4	0.4	1.7	0.2	0.3	0.0	0.0	0.0	0.0	*Bacteria;Firmicutes;Clostridia;Clostridiales;Ruminococcaceae;;;*	56.3	WP_177531663.1
OTU_23	0.0	0.0	0.1	0.0	0.0	0.0	0.0	0.0	0.0	0.0	0.0	0.0	*Bacteria;Bacteroidetes;Bacteroidia;Bacteroidales;Bacteroidaceae;Bacteroides;;*	100.0	WP_104437172.1
OTU_30	0.0	0.0	0.0	0.0	0.0	0.0	0.1	0.0	0.0	0.0	0.0	0.0	*Bacteria;Firmicutes;Clostridia;Clostridiales;Clostridiaceae;;;*	67.9	WP_045175321.1
OTU_32	0.0	0.0	0.0	0.0	0.0	0.0	1.1	0.0	0.0	0.0	0.0	0.0	*Bacteria;Firmicutes;Clostridia;Clostridiales;Clostridiaceae;;;*	85.3	WP_139348778.1
OTU_16	0.0	0.0	0.0	0.0	0.0	0.0	0.2	0.0	0.0	0.0	0.0	0.0	*;;;;;;;*	100.0	WP_101299407.1
OTU_4	0.0	0.0	0.0	0.0	0.0	0.0	0.0	0.0	0.2	0.0	0.1	0.0	*Bacteria;Firmicutes;Clostridia;Clostridiales;Hungateiclostridiaceae;Hungateiclostridium;;*	100.0	WP_003512026.1
OTU_7	0.0	0.0	0.0	0.0	0.0	0.0	0.0	0.0	0.0	0.0	0.0	0.0	*Bacteria;Firmicutes;Clostridia;Clostridiales;Hungateiclostridiaceae;;;*	100.0	WP_069194403.1
OTU_6	0.0	0.0	0.0	0.0	0.0	0.0	0.0	0.0	0.0	0.0	0.0	0.0	*Bacteria;Firmicutes;Clostridia;Clostridiales;Hungateiclostridiaceae;;;*	89.4	WP_043582027.1
OTU_12	0.0	0.1	0.0	0.1	0.0	0.0	0.0	0.0	0.1	0.0	0.0	0.0	*Bacteria;Firmicutes;Clostridia;Clostridiales;Hungateiclostridiaceae;;;*	79.0	WP_003516749.1
OTU_9	0.0	0.0	0.0	0.0	0.0	0.0	36.0	32.7	0.0	0.0	0.0	0.0	*Bacteria;Firmicutes;Clostridia;Clostridiales;Clostridiaceae;;;*	74.1	WP_052659889.1
OTU_14	0.0	0.0	0.0	0.0	0.0	0.0	25.7	26.5	0.0	0.0	0.0	0.0	*Bacteria;Firmicutes;Clostridia;Clostridiales;Clostridiaceae;;;*	76.7	WP_052659889.1

Supplementary Material Script S1: GenPept2txid.py

```
#This python script extracts txid from GenPeptt files.
from Bio import SeqIO
records = SeqIO.parse("sequence.gp","gb")
for record in records:
    for feature in record.features:
        if feature.type == 'source':
            try:
                taxid = feature.qualifiers["db_xref"][0].strip('taxon:')
                print("txid"+taxid)
            except:
                continue

input("Press Enter to exit...")
```

Supplementary Material Script S2: GenBank2Features.py

```
#This python script extracts txid, organism name, nucleotide accession number of the genome assembly, protein accession number of the GH48 module containing protein, corresponding nucleotide sequence and amino acid sequence from GenBank files
from Bio import SeqIO
genbankfile_index = 'genbankfile_index.txt'
with open(genbankfile_index) as f:
    genbank_files = f.read().split('\n')

protein_list = 'features_list.txt'
with open(protein_list) as f:
    proteins = f.read().split('\n')
for file in genbank_files:
    in_handle = open(file, "r")
    for record in SeqIO.parse(in_handle, "genbank"):
        for feature in record.features:
            try:
                if feature.type == 'source':
                    taxid = feature.qualifiers["db_xref"][0].strip('taxon:')
                if feature.type == 'source': #extract name of organism
```

```
                    name = feature.qualifiers['organism'][0]
                if feature.type == 'CDS':
                    product = feature.qualifiers['product'][0]
                if feature.type == 'CDS': #extract CDS
                    prot_id = feature.qualifiers['protein_id'][0]
                    for protein in proteins:
                        if prot_id == protein:
                            target_feature = feature
                            DNA_seq = target_feature.extract(record).seq
                            AA_seq = feature.qualifiers['translation'][0]
                            result = (taxid + '\t' + name + '\t' + product + '\t' + record.id + '\t' + str(DNA_seq) + '\t' + prot_id + '\t' + AA_seq + '\t' + str(RNA_seq) +'\n')
                            print(result)

            except:
                continue
input("Press Enter to exit...")
```

Supplementary Material Script S3: GenPept216S.py

```
#This python script extracts 16S rDNA sequence from GenBank files
from Bio import SeqIO
#Load file_index.txt as an index
genbankfile_index = 'genbankfile_index.txt'
with open(genbankfile_index) as f:
    genbank_files = f.read().split('\n')
for file in genbank_files:
    in_handle = open(file, "r")
    for record in SeqIO.parse(in_handle, "genbank"):
        for feature in record.features:
            try:
                if feature.type == 'source':
                    name = feature.qualifiers['organism'][0]
                if feature.type == 'source':
                    taxid = feature.qualifiers["db_xref"][0].strip('taxon:')
                if feature.type == 'rRNA':
                    product = feature.qualifiers['product'][0]
                    if any([product == '16S ribosomal RNA', product == '16Sa ribosomal RNA']):
                        target_feature = feature #pass selected feature to a variable
                        RNA_seq = target_feature.extract(record).seq
                        result = (taxid + '\t' + name + '\t' + product + '\t' + record.id + '\t' + str(RNA_seq) + '\n')
#result output format
                        print(result)
            except:
                continue
input("Press Enter to exit...")
```
